# Imidazole propionate is increased in diabetes and associated with dietary patterns and altered microbial ecology

**DOI:** 10.1038/s41467-020-19589-w

**Published:** 2020-11-18

**Authors:** Antonio Molinaro, Pierre Bel Lassen, Marcus Henricsson, Hao Wu, Solia Adriouch, Eugeni Belda, Rima Chakaroun, Trine Nielsen, Per-Olof Bergh, Christine Rouault, Sébastien André, Florian Marquet, Fabrizio Andreelli, Joe-Elie Salem, Karen Assmann, Jean-Philippe Bastard, Sofia Forslund, Emmanuelle Le Chatelier, Gwen Falony, Nicolas Pons, Edi Prifti, Benoit Quinquis, Hugo Roume, Sara Vieira-Silva, Tue H. Hansen, Helle Krogh Pedersen, Christian Lewinter, Nadja B. Sønderskov, The MetaCardis Consortium, Renato Alves, Chloe Amouyal, Ehm Astrid Andersson Galijatovic, Olivier Barthelemy, Jean-Paul Batisse, Magalie Berland, Randa Bittar, Hervé Blottière, Frederic Bosquet, Rachid Boubrit, Olivier Bourron, Mickael Camus, Dominique Cassuto, Julien Chilloux, Cecile Ciangura, Luis Pedro Coelho, Jean-Philippe Collet, Maria-Carlota Dao, Morad Djebbar, Angélique Doré, Line Engelbrechtsen, Soraya Fellahi, Leopold Fezeu, Sebastien Fromentin, Philippe Giral, Jens Peter Gøtze, Agnes Hartemann, Jens Juul Holst, Serge Hercberg, Gerard Helft, Malene Hornbak, Jean-Sebastien Hulot, Richard Isnard, Sophie Jaqueminet, Niklas Rye Jørgensen, Hanna Julienne, Johanne Justesen, Judith Kammer, Nikolaj Krarup, Mathieu Kerneis, Jean Khemis, Nadja Buus Kristensen, Michael Kuhn, Véronique Lejard, Florence Levenez, Lea Lucas-Martini, Robin Massey, Nicolas Maziers, Jonathan Medina-Stamminger, Gilles Montalescot, Sandrine Moutel, Laetitia Pasero Le Pavin, Christine Poitou, Francoise Pousset, Laurence Pouzoulet, Sebastien Schmidt, Lucas Moitinho-Silva, Johanne Silvain, Nataliya Sokolovska, Sothea Touch, Mathilde Svendstrup, Timothy Swartz, Thierry Vanduyvenboden, Camille Vatier, Stefanie Walther, Lars Køber, Henrik Vestergaard, Torben Hansen, Jean-Daniel Zucker, Pilar Galan, Marc-Emmanuel Dumas, Jeroen Raes, Jean-Michel Oppert, Ivica Letunic, Jens Nielsen, Peer Bork, S. Dusko Ehrlich, Michael Stumvoll, Oluf Pedersen, Judith Aron-Wisneswky, Karine Clément, Fredrik Bäckhed

**Affiliations:** 1 Wallenberg Laboratory, Department of Molecular and Clinical Medicine and Sahlgrenska Center for Cardiovascular and Metabolic Research, University of Gothenburg, 413 45 Gothenburg, Sweden; 2 Department of Medicine, Sahlgrenska University Hospital, Gothenburg, Sweden; 3 INSERM, Nutrition and Obesities; Systemic Approaches (NutriOmics), Sorbonne Université, Paris, France; 4 Assistance Publique Hôpitaux de Paris, Pitie-Salpêtrière Hospital, Nutrition department, CRNH Ile de France, Paris, France; 5 Integromics Unit, Institute of Cardiometabolism and Nutrition, 75013 Paris, France; 6 Medical Department III - Endocrinology, Nephrology, Rheumatology, University of Leipzig Medical Center, Leipzig, Germany; 7 Novo Nordisk Foundation Center for Basic Metabolic Research, Faculty of Health and Medical Sciences, University of Copenhagen, Blegdamsvej 3B, 2200 Copenhagen, Denmark; 8 Assistance Publique Hôpitaux de Paris, Clinical Investigation Center Paris East, 75013 Paris, France; 9 Assistance Publique Hôpitaux de Paris, Biochemistry and Hormonology Department, Tenon Hospital, 75020 Paris, France; 10 Experimental and Clinical Research Center, A Cooperation of Charité-Universitätsmedizin and the Max-Delbrück Center, Berlin, Germany; 11 Micalis Institute, INRA, AgroParisTech, Université Paris-Saclay, Paris, France; 12 Laboratory of Molecular Bacteriology, Department of Microbiology and Immunology, Rega Institute, KU Leuven Leuven, Belgium; 13 Center for Microbiology, VIB Leuven, Belgium; 14 Unité de Modélisation Mathématique et Informatique des Systèmes Complexes, UMMISCO, 93143 Bondy, France; 15 Sorbonne Paris Cité Epidemiology and Statistics Research Centre (CRESS), U1153 Inserm, U1125, Inra, Cnam, University of Paris 13, Nutritional Epidemiology Research Team (EREN), 93017 Bobigny, France; 16 Computational and Systems Medicine, Department of Metabolism, Digestion and Reproduction, Faculty of Medicine, Imperial College London, London, SW7 2AZ UK; 17 Genomic and Environmental Medicine, National Heart & Lung Institute, Faculty of Medicine, Imperial College London, London, SW3 6KY UK; 18 Biobyte Solutions GmbH, Bothestr. 142, 69117 Heidelberg, Germany; 19 Department of Biology and Biological Engineering, Chalmers University of Technology, SE41128 Gothenburg, Sweden; 20 Structural and Computational Biology, European Molecular Biology Laboratory, Heidelberg, Germany; 21 Molecular Medicine Partnership Unit, University of Heidelberg and European Molecular Biology Laboratory, Heidelberg, Germany; 22 Department of Clinical Physiology, Region Västra Götaland, Sahlgrenska University Hospital, Gothenburg, Sweden; 23 Assistance Publique Hôpitaux de Paris, Diabetes Department, Pitie-Salpêtrière Hospital, Paris, France; 24 Assistance Publique Hôpitaux de Paris, Cardiology Department, Pitie-Salpêtrière Hospital, Paris, France; 25 INRAE, Metagenopolis, Université Paris-Saclay, Jouy en Josas, France; 26 Assistance Publique Hôpitaux de Paris, Pitie-Salpêtrière Hospital, Biochemistry Department of Metabolic Disorders, Paris, France; 27 Institute of Science and Technology for Brain-Inspired Intelligence, Fudan University, Shanghai, China; 28 Centre de Recherche Saint-Antoine, Sorbonne Université-INSERM UMR-S 938, IHU ICAN, Paris, France; 29 Assistance Publique Hôpitaux de Paris, Endocrinology Department, Pitie-Salpêtrière Hospital, Paris, France; 30 Department of Clinical Biochemistry, University of Copenhagen, Rigshospitalet, Copenhagen, Denmark; 31 Assistance Publique Hôpitaux de Paris, Department of Pharmacology, Pitie-Salpêtrière Hospital, NICO Cardio-oncology Program, CIC-1421, INSERM, Sorbonne Université, Paris, France; 32 PARCC, INSERM, Université de Paris, Paris, France; 33 Assistance Publique Hôpitaux de Paris, Hôpital Européen Georges-Pompidou, CIC1418 and DMU CARTE, Paris, France; 34 Integrative Phenomics, Paris, France

**Keywords:** Microbiology, Endocrinology

## Abstract

Microbiota-host-diet interactions contribute to the development of metabolic diseases. Imidazole propionate is a novel microbially produced metabolite from histidine, which impairs glucose metabolism. Here, we show that subjects with prediabetes and diabetes in the MetaCardis cohort from three European countries have elevated serum imidazole propionate levels. Furthermore, imidazole propionate levels were increased in subjects with low bacterial gene richness and Bacteroides 2 enterotype, which have previously been associated with obesity. The Bacteroides 2 enterotype was also associated with increased abundance of the genes involved in imidazole propionate biosynthesis from dietary histidine. Since patients and controls did not differ in their histidine dietary intake, the elevated levels of imidazole propionate in type 2 diabetes likely reflects altered microbial metabolism of histidine, rather than histidine intake per se. Thus the microbiota may contribute to type 2 diabetes by generating imidazole propionate that can modulate host inflammation and metabolism.

## Abstract (web summary)

Gut microbial metabolism of nutrients contributes to metabolic diseases, and the histidine metabolite imidazole propionate (ImP) is produced by type 2 diabetes (T2D) associated microbiome. Here the authors report that circulating ImP levels are increased in subjects with prediabetes or T2D in three European populations, and this increase associates with altered gut microbiota rather than dietary histidine.

## Introduction

Type 2 diabetes is a metabolic and societal disease that is associated with an altered gut microbiome^1–6^, characterized by a lower abundance of butyrate-producing bacteria^1,2^. Fecal microbiota transfer experiments in humans have demonstrated that gut microbiota can directly affect insulin sensitivity providing causal evidence that gut microbiota can contribute to disease development^7,8^. Diet strongly affects the microbial composition and provides a substrate for microbial enzymes generating metabolites, which can modulate host physiology^9^. Since the microbiome differs between ethnicities and different geographical regions^2,10,11^, gut microbiome-derived metabolites might be more conserved biomarkers than specific taxa. Furthermore, metabolites can provide mechanistic insights that may lead to the development of new therapeutic strategies for clinical management of patients with impaired glucose metabolism^12,13^.

Accumulating data suggest that microbial metabolism of dietary components contributes to cardiometabolic diseases^14–17^, but the full appreciation of the interaction between diet and the microbiome in generating such metabolites is still scarce. Some bacterial metabolites such as secondary bile acids^18^, short-chain fatty acids^19^, branched-chain amino acids^15^, and trimethylamines have attracted significant interest in cardiometabolic diseases^20^. We recently identified that imidazole propionate (ImP) is produced by type 2 diabetes associated microbiome through alternative metabolism of histidine, which induces impaired glucose metabolism by activating the p38γ-mTOR1-S6K1 signaling^16,21^.

Here we examine ImP serum levels in a large European multicentric cohort (MetaCardis), from three different European countries, consisting of subjects with different severity of impaired glucose metabolism demonstrating that ImP is increased in subjects with prediabetes and type 2 diabetes. Furthermore, we extend previous studies^16,21^ to demonstrate that ImP is associated with, inflammation, altered microbiome, dietary habits, but not histidine intake.

## Results

### Serum ImP is increased in pre- and type 2 diabetes

The patients with type 2 diabetes in the MetaCardis cohort were slightly older, with a higher proportion of non-Caucasian males compared with healthy individuals and subjects with prediabetes. Patients had an impaired metabolic profile [higher body mass index (BMI) and waist/hip ratio, glucose, insulin, HbA1c, and lipid profiles], while there were no significant differences in kidney function (Table 1).

**Table 1:** Clinical and biochemical features of the MetaCardis cohort.

	Healthy controls	Prediabetes	Type 2 diabetes	*P*-value
*N*	539	654	765	–
Center of enrollment (%)				
France (*n* = 835)	21.4	34.0	44.6	<0.001
Germany (*n* = 587)	34.4	19.3	46.3	
Denmark (*n* = 536)	29.5	47.9	22.6	
Age (years)	53 (39–63)	59 (51–65)	62 (55–67)	<0.001
Male (%)	39	52.6	56.3	<0.001
Non-Caucasian ethnicity (%)	7.2	10.2	20.5	<0.001
Body Mass Index (kg/m*2)	25.9 (22.6–38.2)	30.4 (25.8–39.1)	31.9 (28.3–36.7)	<0.001
Waist/hip ratio	1 (1–1.3)	1.1 (1–1.3)	1.1 (1–1.2)	<0.001
Glucose (nM)	5 (4.7–5.3)	5.7 (5.3–6)	7.5 (6.3–8.9)	<0.001
Insulin (mUI)	6.9 (4.4–11.3)	9.9 (6.6–14.5)	12.5 (8.7–21)	<0.001
HbA1c (%)	5.4 (5.2–5.5)	5.8 (5.6–6)	6.8 (6.3–7.6)	<0.001
Triglycerides (mM/l)	1 (0.7–1.4)	1.2 (0.9–1.6)	1.5 (1.1–2.2)	<0.001
Creatinine clearance (ml/min)	87.5 (77.4–100)	86.4 (75–97.7)	86.4 (74.2–100.2)	0.206
Medications (%)				
Any anti-diabetic treatment	0	0	81.7	<0.001
Metformin	0	0	69.8	<0.001
Any lipid-lowering treatment	21.5	36.2	51.5	<0.001
Statins	21.2	33.9	48.1	<0.001

Data are presented as median and interquartile range or as a percentage. *P*-values for continuous variables were calculated using linear regression. *P*-values for categorical variables were calculated using the Fisher test.

**Fig. 1:**
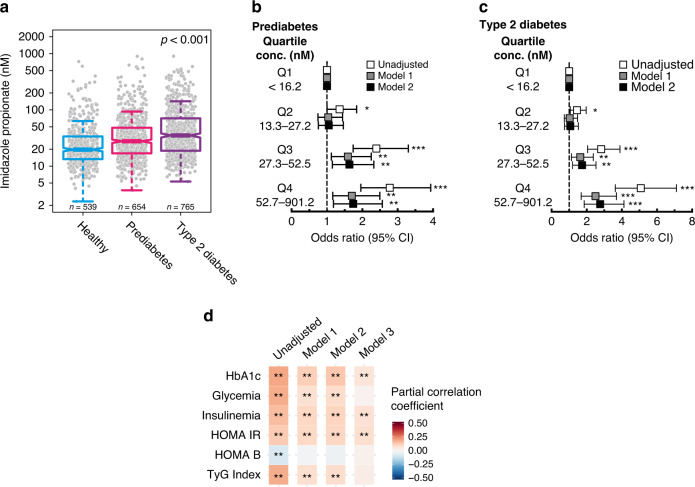
Imidazole propionate is increased in subjects with type 2 diabetes. **a** Serum levels of imidazole propionate in healthy subjects (*n* = 539), subjects with prediabetes (*n* = 654), and with type 2 diabetes (*n* = 765). *P*-values were calculated with linear regression. Data are represented as boxplots: middle line is the median, the lower and upper hinges are the first and third quartiles, the upper whisker extends from the hinge to the largest value no further than 1.5× the interquartile range (IQR) from the hinge, and the lower whisker extends from the hinge to the smallest value at most 1.5× IQR of the hinge. Gray dots are single data points. **b**, **c**. Multinomial logistic regression for prediabetes and type 2 diabetes vs. healthy controls according to imidazole propionate quartiles. Odds ratios (OR) were calculated using the lowest quartile of imidazole propionate (Q1) as reference. Model 1 OR was adjusted for age, gender, body mass index (BMI), and ethnicity. Model 2 OR was adjusted for model 1 plus creatinine clearance. Squares represent OR and the upper and lower whisker the 95% confidence intervals (CI), raw data are presented in Supplementary Table 1. *P < 0.05, **P < 0.01, ***P < 0.001 **d**. Correlation matrix for imidazole propionate and glycated hemoglobin (HbA1c), glycemia, insulinemia, homeostatic model assessment of insulin resistance (HOMA-IR), updated HOMA model for beta-cell function (HOMA-B), and the triglyceride and glucose (TyG) index. Pearson partial correlation coefficients and *P*-values were calculated using partial correlations adjusted for Model 1: age, gender, body mass index, and ethnicity. Model 2: Model 1 plus creatinine clearance, Model 3: Model 2 plus diabetes status. False discovery rate (FDR) adjusted *P < 0.05, **P < 0.01. See also Supplementary Table 2. Source data are provided as a Source Data file.

ImP serum levels were significantly higher in subjects with pre- and type 2 diabetes compared with healthy controls (Fig. 1a), with no impact of subject ethnic background (Supplementary Fig. 1a). Similar observations were made in all enrollments centers (Supplementary Fig. 1b). As compared to subjects in the lowest quartile of ImP levels, those in the highest quartile had a significantly higher risk of having prediabetes [odds ratio (OR) 1.75; 95% confidence interval (CI) 1.18–2.57; *P* = 0.006] and type 2 diabetes [OR 2.76, 95%; CI 1.86–4.12, *P* < 0.001; Fig. 1b, c, Supplementary Table 1, after adjusting for traditional risk factors (Model 1: age, gender, BMI, ethnicity) and for kidney function (Model 2: Model 1 + creatinine clearance)].

We next examined associations between ImP levels and markers of glucose and lipid metabolism as well as for surrogates of insulin resistance. ImP correlated positively with fasting HbA1c, glycemia, insulinemia, HOMA-IR, and triglyceride-glucose index and negatively with HOMA-B. These results indicate a link between circulating ImP and impaired glucose metabolism profiles. Importantly, correlations remained significant after adjustment for known traditional risk factors (Model 1: age, gender, BMI and ethnicity), for kidney function (Model 2: Model 1 + creatinine clearance), and even the presence of type 2 diabetes (Model 3: Model 2 + diabetes status, Fig. 1d, Supplementary Table 2).

To investigate if ImP also was associated with the dynamic assessment of glucose metabolism, we performed further analyses on a subpopulation (*n* = 586) where oral glucose tolerance tests (OGTT) were performed. Subject stratification based on ImP quartiles revealed that elevated ImP levels were associated with increased glucose, insulin, and C-peptide levels 2 hours after OGGT, translating to reduced Stumvoll sensitivity index (Supplementary Table 3).

All together, serum ImP is increased in pre- and type 2 diabetes and associates with markers of impaired glucose metabolism independently of diabetes status.

### ImP is associated with diabetes treatment and co-morbidities

Metformin is the first line of treatment for type 2 diabetes and has a profound effect on microbiota composition and function^22,23^. ImP can alter the glucose-lowering effects of metformin treatment^21^. Thus, we evaluated the effects of metformin and other anti-diabetic drugs on ImP levels (Supplementary Fig. 1c). Patients with metformin-treated type 2 diabetes had higher levels of ImP compared with those without any treatment. This could be due to the altered microbiome following metformin treatment^22,23^ or reflecting a more severe disease phenotype that required polypharmacy. Indeed, subjects treated with insulin and additional anti-diabetic drugs also had increased ImP levels. When we performed a sub-analysis including only subjects naïve for anti-diabetic treatments, subjects with type 2 diabetes displayed significantly increased levels of ImP [28.1 nM (16.1–59.2) median and interquartile range; *n* = 140] compared to subjects with prediabetes [27.8 nM (17–49.3) median and interquartile range; *n* = 654] or normal glucose tolerance [19.7 nM (13.2–33.9) median and interquartile range; *n* = 359] (*P* = 0.028, linear regression after adjustment for age, gender, BMI, ethnicity, and creatinine clearance).

When we performed a sub-analysis for the presence of cardiovascular diseases (CVD) we could observe that subjects with CVD had significantly increased levels of ImP [36.7 nM (20.5–69.1) median and interquartile range; *n* = 390] compared to the subjects without CVD [25.2 nM (15.2–47.7) median and interquartile range; *n* = 1568] (*P* < 0.001, linear regression after adjustment for age, gender, BMI, ethnicity, diabetes status, and creatinine clearance) which requires further investigation.

### ImP serum levels are associated with an altered microbiome

**Fig. 2:**
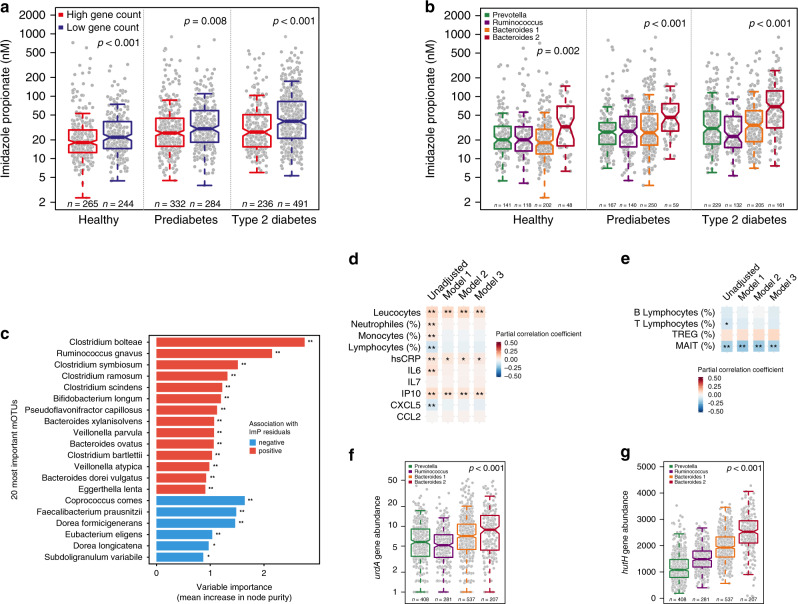
Imidazole propionate is associated with a pro-inflammatory microbiota. Serum levels of imidazole propionate (ImP) in healthy subjects (*n* = 509), subjects with prediabetes (*n* = 616), and with type 2 diabetes (*n* = 727) according to **a** bacterial gene count and **b** enterotypes. *P-*values were calculated with linear regression adjusted for age, gender, BMI, ethnicity, and creatinine clearance. **c** Random forest for the 20 most significant mOTUs correlated with ImP residuals, after adjustment for age, gender, BMI, ethnicity, creatinine clearance, and diabetes status. FDR adjusted *P*-value of spearman correlation between taxa and imp residuals *P < 0.05, **P < 0.01. See also Supplementary Table 4. **d** Partial correlation matrix for ImP serum levels and serum leucocytes count (10^9^/l), neutrophils (%), monocytes (%), lymphocytes (%), C-reactive protein (CRP), Interleukin 6 (IL-6), Interleukin 7 (IL-7), Interferon gamma-induced protein 10 (IP-10), C-X-C motif chemokine 5 (CXCL5), chemokine (C-C motif) ligand 2 (CCL2). Pearson partial correlation coefficients and *P*-values were calculated using partial correlations adjusted for Model 1: age, gender, body mass index, and ethnicity. Model 2: Model 1 plus creatinine clearance, Model 3: Model 2 plus diabetes status. *P < 0.05, **P < 0.01, ***P < 0.001. See also Supplementary Table 5. **e** Partial correlation matrix in a subgroup of patients (*n* = 439) between serum ImP and circulating B- and T lymphocytes (%), regulatory T cells (TREG, %) and mucosal-associated invariant T cell (MAIT, %). Partial correlation coefficients (Pearson for all variables except for MAIT cells for which Spearman coefficient was used since variable distribution remained skewed despite log-transformation) and *P*-values were calculated using partial correlations for Model 1: age, gender, body mass index, and ethnicity. Model 2: Model 1 plus creatinine clearance, Model 3: Model 2 plus diabetes status. *P < 0.05, **False discovery rate (FDR) adjusted *P* < 0.05. See also Supplementary Table 6. Relative abundances of *urdA* gene (**f**) and *hutH* (**g**) according to enterotype. *P-*values were calculated with linear regression adjusted for age, gender, BMI, and ethnicity. For **a**, **b**, **f**, **g** data are represented as boxplots: middle line is the median, the lower and upper hinges are the first and third quartiles, the upper whisker extends from the hinge to the largest value no further than 1.5× the interquartile range (IQR) from the hinge and the lower whisker extends from the hinge to the smallest value at most 1.5× IQR of the hinge. Gray dots are single data points. Source data are provided as a Source Data file.

Since low microbiome gene count is associated with obesity, insulin resistance, and dyslipidemia^24^, we next investigated if increased circulating ImP levels are associated with an altered microbiome. Thus, the study population was stratified into high and low gene count (threshold: 607,000 genes) and observed that subjects with low gene count had higher circulating ImP levels compared to those with high gene count, independently of diabetes status (Fig. 2a). We observed a significant negative correlation between ImP residuals (ImP levels adjusted for age, gender, BMI, ethnicity, and creatine clearance) and gene count independently of diabetes status. Interestingly, the effect size was even stronger for type 2 diabetes (*R* = −0.31, *P* < 0.001) compared with prediabetes and healthy subjects (Supplementary Fig. 2a).

The human gut microbiome can be separated into community types, also known as enterotypes^25^. We next analyzed if ImP levels were associated with specific enterotypes and observed that subjects with Bacteroides 2 enterotype had significantly increased serum ImP levels compared with other enterotypes (Fig. 2b). This enterotype has been linked to low gene richness and low bacterial cell load as well as impaired metabolism^26,27^ and pro-inflammatory conditions such as inflammatory bowel disease^28,29^. We next investigated if specific mOTUs were associated with ImP levels using a random forest approach. We observed that *Clostridium bolteae*, *Clostridium symbiosum,* and *Ruminococcus gnavus* were the most important mOTUs positively associated with ImP after adjustment for age, gender, BMI, ethnicity, creatine clearance, and diabetes status (Fig. 2c and Supplementary Table 4). Increased abundance of these bacteria has previously been reported in subjects with metabolic diseases such as type 2 diabetes and prediabetes^1,5^ as well as in subjects with inflammatory bowel disease (IBD)^30^. In contrast, other bacteria with anti-inflammatory capacity^31,32^, such as *Faecalibacterium prausnitzii* were negatively associated with ImP serum levels. Taken together these data suggest that ImP serum levels are linked to a pro-inflammatory microbiota composition, in agreement with that ImP initially was identified to be increased in subjects with gut inflammation^33^.

### ImP is associated with systemic inflammation

Next, we explored whether ImP levels were associated with inflammatory serum markers in the population and observed that serum ImP levels were positively correlated with serum markers of inflammation [total leucocytes count, high sensitive C-reactive protein (hs-CRP), interferon gamma-induced protein 10 (IP-10)] after correction for known traditional risk factors (Model 1: age, gender, BMI, ethnicity), for kidney function (Model 2: Model 1 + creatinine clearance), and for the presence of type 2 diabetes (Model 3: Model 2 + diabetes status) (Fig. 2d and Supplementary Table 5). Moreover, by examining a subpopulation (*n* = 439) from the MetaCardis cohort with peripheral lymphocytes characterization^34^, we observed a significant negative correlation between ImP levels and circulating mucosal-associated invariant T cells (MAIT, Fig. 2e and Supplementary Table 6), which have innate effector-like qualities defending against microbial infections. Interestingly, the reduction in peripheral MAIT cells has been linked with metabolic diseases and obesity, and with cardiometabolic disease progression in the MetaCardis population^34^. Taken together, low gene count microbiome and Bacteroides 2 enterotype are associated with increased circulating ImP levels that may contribute to type 2 diabetes by promoting low-grade inflammation.

### Microbial metabolism of histidine

To gain further understanding of how the microbiota metabolizes histidine to ImP we analyzed the abundance of the *hutH* gene encoding histidine ammonia lyase and of *urdA*, the gene encoding urocanate reductase. A major challenge for assessing enzyme specificity is that several enzymes with homologous sequences may have different substrate specificity. However, *urdA* can be identified based on amino acids in the FAD-binding domains in the active site^16^. *urdA* is a low abundant gene whereas *hutH* is more prevalent and observed in 201 metagenomic species (Supplementary Table 7). After correction for age, gender, BMI, ethnicity, and creatinine clearance both *hutH* and *urdA* abundances were increased according to diabetes status and ImP quartiles (Supplementary Fig. 2b–e). As expected, both *urdA* and *hutH* abundances were increased in the Bacteroides 2 enterotype (Fig. 2f, g). In agreement with the association between the increased abundance of *hutH* and *urdA* with Bacteroides 2 enterotype, we also observed negative correlations between these genes and gene richness (rho = −0.41, *P* < 0.001 and rho = −0.25, *P* < 0.001 for *hutH* and *urdA*, respectively) (Supplementary Fig. 2f, g).

### Unhealthy dietary patterns are associated with serum ImP

**Fig. 3:**
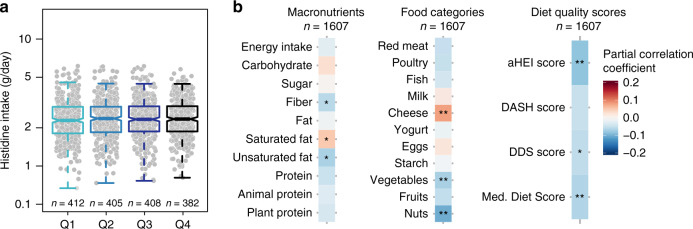
Imidazole propionate is associated with an unhealthy diet, but not histidine intake. **a** Dietary histidine intake according to quartiles of imidazole propionate. *P-*values were calculated with linear regression adjusted for age, gender, body mass index, ethnicity, diabetes status, creatinine clearance, daily energy intake (kcal/day), and enrollment center. Data are represented as boxplots: middle line is the median, the lower and upper hinges are the first and third quartiles, the upper whisker extends from the hinge to the largest value no further than 1.5× the interquartile range (IQR) from the hinge, and the lower whisker extends from the hinge to the smallest value at most 1.5× IQR of the hinge. Gray dots are single data points. **b** Correlation matrix for imidazole propionate serum levels and macronutrients, food categories and dietary scores [the alternate Healthy Eating Index (aHEI), Dietary Approaches to Stop Hypertension (DASH) score, dietary diversity score (DDS) and Mediterranean diet score]. Pearson partial correlation coefficients and *P*-values were calculated using partial correlations adjusted for age, gender, body mass index, ethnicity, diabetes status, creatinine clearance, daily energy intake (kcal/day), and enrollment center. *P < 0.05, **False discovery rate (FDR) adjusted *P* < 0.05. See Supplementary Table 8. Source data are provided as a Source Data file.

Histidine is a precursor of ImP, accordingly, we evaluated the daily dietary histidine intake in our study population. Based on food-frequency questionnaire records, we did not observe any significant differences in histidine intake when the population was stratified according to ImP quartiles (Fig. 3a). Accordingly, we did not observe differences in circulating histidine levels in controls and subjects with type 2 diabetes (*n* = 1895; *P* = 0.78). Next, we evaluated the full spectrum of nutrient intake and identified a significant positive correlation between ImP and saturated fat intake (driven by high cheese intake) and negative correlations with fiber and unsaturated fat intake (driven by reduced intake of vegetables and nuts, Fig. 3b). Moreover, we examined more broadly dietary patterns as assessed by different indexes/scores [the alternate Healthy Eating Index (aHEI), Dietary Approaches to Stop Hypertension (DASH) score, Dietary Diversity Score (DDS) and Mediterranean diet score]^35–37^. ImP serum levels correlated negatively with aHEI, DSS, and Mediterranean diet scores, after correction for age, gender, BMI, ethnicity, center (country), daily energy intake, creatinine clearance, and diabetes status. It is important to note that the effect size for these associations is relatively small but overall indicates that an unhealthy diet was associated with increased levels of ImP (Fig. 3b and Supplementary Table 8).

## Discussion

The gut microbiome produces a myriad of metabolites that modulate insulin signaling^38^, and we recently identified that the histidine metabolite ImP is produced by the microbiome of subjects with type 2 diabetes^16^. Here we demonstrated that ImP is increased in patients with type 2 diabetes in a European multicentric cohort of subjects from different origins. In contrast, to the microbiota that differs between countries^2^, regions within a country^11^, or even between ethnicities within a city^10^, we observed that ImP levels are increased in type 2 diabetes in French, Danes, and Germans in addition to Dutch^16^, Swedes^16^, and Chinese^39^ as previously reported. Metabolites, such as ImP, may then provide a more relevant indicator of an altered microbial function across populations than metagenomic sequencing per se. Gut microbiome stratification revealed that the Bacteroides 2 enterotype, and reduced gene richness, were associated with increased ImP. Low gene richness has previously been associated with low-grade inflammation, metabolic and inflammatory disorders^24^.

Here we observed that ImP was associated with increased levels of pro-inflammatory cytokine and a reduced number of MAIT cells, emanating from the gut mucosa. This is consistent with the fact that ImP is a potent activator of the MAP-kinase p38γ^16^. ImP was originally identified in subjects with gastrointestinal inflammation and is also associated with inflammatory bowel disorders^33^. We have previously demonstrated that type 2 diabetes is associated with increased inflammatory tone in the gut^40^, which is consistent with increased ImP levels. Thus one can speculate that the increased levels of ImP in the gut may contribute to elevated inflammatory tone in the mucosal lining.

Here we demonstrate that ImP levels were associated with a low abundance of microbial diversity and Bacteroides 2 enterotype, which previously have been linked to obesity^27^ and inflammatory bowel diseases^28^. These findings are consistent with the fact that subjects with type 2 diabetes have an altered microbiota including reduced diversity and reduced abundance of butyrate-producing bacteria including *F. prauznitzii*^1–6^. Furthermore, recent studies demonstrated that ImP can predict alpha-diversity in humans^13^, which also is associated with type 2 diabetes^12^. We observed that an unhealthy diet, but not histidine intake, was associated with increased ImP levels. These findings suggest that rather than being affected by histidine as a substrate, increased ImP production may, at least in part, be the result of an unhealthy diet changing microbial environment and its capacity to produce ImP.

Several reasons could explain the absence of link observed between FFQ-extrapolated histidine intake and ImP levels: (i) ImP production by bacteria requires specific bacterial enzymes^16^ and therefore the limiting factor may not be the availability of the substrate (histidine) but the presence of the bacteria with the capacity to produce ImP; (ii) histidine degradation is tightly regulated to maintain sufficient intracellular pools of histidine and Hut enzymes are not formed at maximal rates unless bacteria are limited in other carbon sources most commonly obtained from fiber^41^; (iii) long term dietary habits are key shaping factors for the gut microbiota and an unhealthy diet poor in fiber and rich in low saturated fats may lead to a dysbiotic microbial environment which will ultimately lead to higher ImP levels^42^; (iv) we cannot exclude that the methods used to capture histidine intake have limited resolution to detect small differences in intake. However, overall, our findings suggest that ImP is not directly linked to dietary histidine intake, but rather an unhealthy diet with reduced intake of fiber and unsaturated fatty acids that results in a dysbiotic microbiome with increased capacity to produce ImP. One limitation of our data is that that we have not used an independent validation cohort to confirm our findings. Further studies using independent cohorts are needed to confirm the role of ImP in type 2 diabetes.

In summary, our data suggest that an unhealthy diet may contribute to an altered microbial community type with increased potential to metabolize dietary histidine to ImP, which in turn contributes to impaired glucose metabolism by activating MAPK signaling leading to degradation of insulin receptor substrate^16^ and inflammatory signaling^43^. Since ImP has been observed to be increased in subjects with glucose intolerance and type 2 diabetes of several origins, personalized dietary recommendation or inhibition of *urdA* might be helpful for reducing circulating ImP levels.

## Methods

### Study population

We examined 1990 subjects from the MetaCardis cohort for whom a serum sample was available but excluded 32 patients due to non-metabolic etiology of cardiovascular diseases (*n* = 25), clear outliers for ImP levels according to Grubb’s test (*n* = 1), non-complete biochemistry data (*n* = 6). Subjects were recruited between 2013 and 2015 in clinical institutions in France (Pitié-Salpêtrière Hospital, Center of Research for Clinical Nutrition (CRNH), Institute of Cardiometabolism And Nutrition (ICAN)), Germany (Integrated Research and Treatment Center (IFB) Adiposity Diseases in Leipzig) and Denmark (Novo Nordisk Foundation Center for Basic Metabolic Research (NNFCBMR) in Copenhagen) for the European project MetaCardis. www.metacardis.net.

Patients with a history of abdominal surgery (other than appendicitis or cholecystectomy), abdominal radiotherapy, digestive cancer or that had received a recent antibiotic treatment (<2 months) were not included. Patients that had participated in the previous cohort-based study were contacted for potential inclusion. A subgroup of healthy control individuals with no signs of obesity or metabolic syndrome were recruited through advertisement and through existing population cohorts. All subjects provided written informed consent and the study was conducted in accordance with the Helsinki Declaration and is registered in clinical trial https://clinicaltrials.gov/show/NCT02059538. The Ethics Committee of each participating country approved the clinical investigation. The study was approved by the Comite de Protection des Personnes (CPP) Ile de France III no. IDRCB2013-A00189-36.

A detailed list of prescribed medications, anthropometric data, clinical history, fecal sample, and a fasting blood sample was obtained at enrollment. Subjects were classified as healthy, prediabetes, or type 2 diabetes. Type 2 diabetes was defined as fasting glycemia ≥7.0 mmol/l and/or 2 h values during the oral glucose tolerance test >11.1 mmol/l and/or hemoglobin A1c (HbA1c, glycated hemoglobin) ≥6.5% (≥48 mmol/mol) and/or use of any anti-diabetic treatment; prediabetes was defined for subjects without type 2 diabetes as fasting glycemia ≥5.6 mmol/l and/or 2 h values in the oral glucose tolerance test ≥7.8 mmol/l and/or hemoglobin A1c (HbA1c, glycated hemoglobin) ≥5.7% (≥39 mmol/mol) according to the American Diabetes Association (ADA) definitions^44^.

### Dietary intake data and diet quality assessment

Dietary data were collected via a food-frequency questionnaire that was adapted to the cultural habits of each of the countries of recruitment. A validation study against repeated 24 h-dietary records among 324 French MetaCardis participants has indicated an acceptable validity^45^. Specifically for this study, histidine intake was calculated based on values concerning the histidine content of selected foods published online in the United States Department of Agriculture (USDA) food composition databases https://ndb.nal.usda.gov/ndb/nutrients/. Food groups were further refined by subdividing the original 22 groups into 37, which were used to calculate the total dietary intake of histidine. Dietary quality scores have been adapted from the scores used in the framework of the multicenter European study EPIC^46–49^.

For each subject, the basal metabolic rate (BMR) was estimated using Harris and Benedict Formula^50^. Subjects with aberrant energy intake declarations defined as <0.5*BMR or >3.5*BMR were excluded from all nutritional analysis (<10% of the subjects with available nutritional data). In total, 1607 subjects were included in the nutritional analysis.

### Biochemical analyses

Blood samples were collected after an overnight fast. Fasting serum glucose, triglycerides, and HbA1c were measured using enzymatic methods. Fasting serum insulin and C-peptide were measured using a chemiluminescence assay (Insulin Architect, Abbott). High-sensitivity C-reactive protein (hs-CRP) was measured using an IMMAGE automatic immunoassay system (Beckman-Coulter) and high-sensitivity interleukin 6 (hs-IL-6) was measured using the Human IL-6 Quantikine HS ELISA Kit (R&D Systems). IFN-γ–induced protein 10 (IP-10), interleukin 7 (IL-7), C-X-C motif chemokine ligand 2 (CXCL2), and 5 (CXCL5) were measured by using a Luminex assay (ProcartaPlex Mix&Match Human 13-plex; eBioscience, San Diego, CA, USA).

### Oral glucose tolerance test

For 586 subjects of the Metacardis cohort without any clinical/laboratory sign of type 2 diabetes and thus naïve of anti-diabetic treatments, at the inclusion visit, an oral 75 g-glucose tolerance test (OGTT) was performed following standard of care. Serum glucose, insulin, and C-peptide were measured at baseline and 120 min after the glucose load.

### Imidazole propionate serum measurements

ImP was quantified using ultra-performance liquid chromatography coupled to tandem mass spectrometry according to previous work. Briefly, serum samples were extracted with 3 volumes of ice-cold acetonitrile containing internal standards (13C3-labeled ImP and urocanate). After derivatization to butyl esters using 5% hydrochloric acid in butanol, the samples were separated on a C18 column using a gradient consisting of water and acetonitrile. Quantification was made using an external calibration curve^16^.

### Flow cytometry analysis

To characterize and quantify immune cells, 100 µl of whole blood was freshly obtained in a subgroup of subjects (*n* = 439) belonging to one of the centers involved in the MetaCardis consortium (France, Pitié-Salpêtrière Hospital, Institute of Cardiometabolism And Nutrition cytometry platform)^34^. Briefly, blood was incubated with FcR blocking reagent (Miltenyi Biotec, Bergisch Gladbach, Germany), red blood cell lysated, and then white blood cells were stained with the following antibodies: Vioblue-anti-human CD3 (clone BW264/56), PE-Vio770-anti-human CD4 (VIT4), PerCP-Vio700-anti-human CD8 (BW135/80), APC-anti-human CD25 (4E3), FITC-anti-human CD127 (MB15-18C9), PE-anti-human CD161 (191B8) from Miltenyi Biotec, and APC-Cy7-anti-human TCR Vα7.2 (3C10) from BioLegend (San Diego, CA, USA). Data acquisition was performed with a MacsQuant Analyzer using MacsQuantify software (Miltenyi Biotec). Data obtained were analyzed with FlowJo 10.1r5 software (Tree Star, Ashland, OR, USA).

### Extraction of fecal genomic DNA and whole-genome shotgun sequencing

Participants collected fecal samples within 24 h before each visit. Samples were either stored immediately at −80 °C or briefly conserved in home freezers, before transport to the laboratory where they were immediately frozen at −80 °C following guidelines^51^. Total fecal DNA was extracted following the International Human Microbiome Standards (IHMS) guidelines (SOP 07 V2 H) and sequenced using ion-proton technology (ThermoFisher Scientific) resulting in 23.3 ± 4.0 million (mean ± SD) 150-bp single-end reads per sample on average. Reads were cleaned using Alien Trimmer (v0.2.4)^39^ in order to remove resilient sequencing adapters and to trim low-quality nucleotides at the 3′ side (quality and length cut-off of 20 and 45 bp, respectively). Cleaned reads were subsequently filtered from human and potential food contaminant DNA (using human genome RCh37-p10, *Bos taurus*, and *Arabidopsis thaliana* with an identity score threshold of 97%). The reads were mapped to the Integrated Gene Catalog (IGC) of 9.9 million genes^52^, with Bowtie 2.2.4. For each read, the best alignment is conserved. Reads mapped to the main reference with at least 95% of identity are conserved for the counting step if they are not mapped against contaminant references with at least 97% of identity. Gene counts were generated using a two-step procedure (called smart shared counting). First, the unique mapped reads (reads mapping to a unique gene from the catalog) were attributed to the corresponding genes. Second, the shared reads (mapping different genes of the catalog) were attributed according to the ratio of their unique mapping counts. Gene abundance tables (built from mapping against the 9.9 M gene catalog) were processed for richness calculation, downsizing, and normalization using the momr R package. In order to reduce technical bias due to variable sequencing depth, Ion-Proton samples were downsized to 10 million reads, and downsized gene abundances were normalized according to Fragments Per Kilobase per Million mapped reads (FPKM) strategy.

### Assessment of gut microbiota characteristics

Metagenomic data were available for 1852 subjects. Abundance for each MGS (metagenomic species) was computed as the mean value of the 50 genes defining a robust centroid of the cluster (if more than 10% of these genes gave positive signals) as proposed^53^ for MGS with >500 genes using momr R package. MGS taxonomical annotation was performed using all genes by sequence similarity using NCBI blastN; a species-level assignment was given if >50% of the genes matched the same reference genome of the NCBI database (November 2016 version) at a threshold of 95% of identity and 90% of gene length coverage. The remaining MGS were assigned to a given taxonomical level from the genus to superkingdom if more than 50% of their genes had the same level of assignment. Microbial gene richness (gene count) was calculated by counting the number of genes that were detected at least once in a given sample, using the average number of genes counted in ten independent rarefaction experiments. Alpha-diversity was measured as gene richness i.e., the average number of genes (meaning at least one read mapped) per sample, and subjects were classified in metagenomic richness status (low or high gene count). Metagenomic richness status defined was defined using the threshold separating the bimodal distributions of gene richness in the healthy control group of the German center (where bimodality is revealed) i.e., <607,000 genes for the low gene count group and ≥607,000 genes for the high gene count group. Enterotyping of the cohort was performed following the Dirichlet Multinomial Mixture (DMM) method using microbial taxons (mOTU) abundance matrix of the entire cohort collapsed at the genus level^29,54,55^.

### *hutH* analyses

To determine the abundance of *hutH*, the genes encoding histidine ammonia-lyase that metabolizes histidine to urocanate, we quantified the abundance of Kyoto Encyclopedia of Genes Genomes (KEGG) ortholog (KO) K01745. To identify the metagenomic species with the functional capacity to degrade histidine to urocanate, we projected K01745 on all MGS > 500 genes.

### urdA analyses

To determine the DNA abundance of *urdA*, the gene encoding urocanate reductase responsible for ImP production, we used the same pipeline as previously^16^ but extend to the latest release of NCBI bacterial genomes which contains 557,951,640 protein-coding genes (as accessed in February 2019) and identified 63,961 potential *urdA* homologs. Exact read mapping was restricted to gene regions containing only 90 nt both down- and up-stream of the FAD active sites of the *urdA* genes based on bowtie 2 with no mismatches and gap opening during reads alignment extracting 12,319 non-redundant sequences for further metagenomic mapping. Of those 12,319 gene regions. In addition, only samples with at least 10 reads mapped and reads with a mapping length larger than 100 bp were included for analysis to ensure that the matched reads cover the active sites. The total reads mapped were then normalized by the sequencing depth in each sample to separate sequences based on true and false urocanate reductases based on the amino acid in position 373. 4760 reads identified as true UrdA without histidine in the 373 position as well as 4968 as with histidine and that position. We have previously shown that histidine in that position prevents urocanate reductase activity^16^.

### Statistical analysis

The updated homeostatic model assessment of insulin resistance, sensitivity, and beta-cell function (HOMA2-IR, HOMA2-S, HOMA2-B), the quantitative insulin sensitivity check index (QUICKI), the triglycerides and glucose index (TyG) and the Stumvoll index were calculated as described previously^56–60^. Estimated glomerular filtration rate (eGFR) was calculated using the Modification of Diet in Renal Disease (MDRD) formula^61^. All nutrient and food group data are expressed as g of intake per day. Diet quality assessed using four previously validated nutritional scores: the alternate Healthy Eating Index (aHEI), Dietary Approaches to Stop Hypertension (DASH) score, dietary diversity score (DDS), and the Mediterranean diet score^35–37,46^. For descriptive statistics, continuous variables were presented in the median and interquartile range. ImP levels were categorized into quartiles in the full analysis cohort of 1958 individuals. Categorical variables were presented as numbers and percent. Analyses were performed using linear regression models as unadjusted or adjusted for confounding factors (see text and figure legends). For nutritional data, a further adjustment was performed when precised on daily total energy intake and center. In quantitative analysis (graphically presented as heatmaps), partial correlations were performed unadjusted or adjusted for confounders. Variables with skewed distributions were logarithmically transformed before entering the models (age, BMI, creatinine clearance, 2 h glucose, insulin, C-peptide, Stumvoll sensitivity index and Imidazole propionate were log-transformed, Shapiro–Wilk test *P* < 0.05). Correlation analysis of gene richness and functional features (*urdA*, *hutH*) were assessed by Spearman’s correlations. The most important mOTUs for the prediction of ImP levels were identified with cross-validated random forest models using ImP residuals adjusted for age, gender, BMI, diabetes status, ethnicity, and creatinine clearance with the randomForest R package. An optimal mtry and max node of 50 for the trees was determined using the mean squared error of ImP residuals in test samples as the outcome. The mean variable importance (using an increase in node purity) was determined with 100-fold cross-validation.

Statistical analyses were carried out using R statistical analysis software version 3.3.2 (http://www.R-project.org/).

### Reporting summary

Further information on research design is available in the Nature Research Reporting Summary linked to this article.

## Supplementary information

Supplementary Information

Reporting Summary

## Data Availability

Gender and center data were removed from source data files in order to maintain participant confidentiality. These data can accessed by a request to Professor Karine Clement.Raw sequencing data used in this study have been deposited in the EMBL-EBI European Nucleotide Archive (ENA) under accession numbers PRJEB37249 and PRJEB38742. For clinical cohort-related questions, contact K.C. Source data are provided with this paper.

## Source data

Source Data
