# Author Correction: Imidazole propionate is increased in diabetes and associated with dietary patterns and altered microbial ecology

**DOI:** 10.1038/s41467-020-20412-9

**Published:** 2020-12-21

**Authors:** Antonio Molinaro, Pierre Bel Lassen, Marcus Henricsson, Hao Wu, Solia Adriouch, Eugeni Belda, Rima Chakaroun, Trine Nielsen, Per-Olof Bergh, Christine Rouault, Sébastien André, Florian Marquet, Fabrizio Andreelli, Joe-Elie Salem, Karen Assmann, Jean-Philippe Bastard, Sofia Forslund, Emmanuelle Le Chatelier, Gwen Falony, Nicolas Pons, Edi Prifti, Benoit Quinquis, Hugo Roume, Sara Vieira-Silva, Tue H. Hansen, Helle Krogh Pedersen, Christian Lewinter, Nadja B. Sønderskov, The MetaCardis Consortium, Renato Alves, Chloe Amouyal, Ehm Astrid Andersson Galijatovic, Olivier Barthelemy, Jean-Paul Batisse, Magalie Berland, Randa Bittar, Hervé Blottière, Frederic Bosquet, Rachid Boubrit, Olivier Bourron, Mickael Camus, Dominique Cassuto, Julien Chilloux, Cecile Ciangura, Luis Pedro Coelho, Jean-Philippe Collet, Maria-Carlota Dao, Morad Djebbar, Angélique Doré, Line Engelbrechtsen, Soraya Fellahi, Leopold Fezeu, Sebastien Fromentin, Philippe Giral, Jens Peter Gøtze, Agnes Hartemann, Jens Juul Holst, Serge Hercberg, Gerard Helft, Malene Hornbak, Jean-Sebastien Hulot, Richard Isnard, Sophie Jaqueminet, Niklas Rye Jørgensen, Hanna Julienne, Johanne Justesen, Judith Kammer, Nikolaj Krarup, Mathieu Kerneis, Jean Khemis, Nadja Buus Kristensen, Michael Kuhn, Véronique Lejard, Florence Levenez, Lea Lucas-Martini, Robin Massey, Nicolas Maziers, Jonathan Medina-Stamminger, Gilles Montalescot, Sandrine Moutel, Laetitia Pasero Le Pavin, Christine Poitou, Francoise Pousset, Laurence Pouzoulet, Sebastien Schmidt, Lucas Moitinho-Silva, Johanne Silvain, Nataliya Sokolovska, Sothea Touch, Mathilde Svendstrup, Timothy Swartz, Thierry Vanduyvenboden, Camille Vatier, Stefanie Walther, Lars Køber, Henrik Vestergaard, Torben Hansen, Jean-Daniel Zucker, Pilar Galan, Marc-Emmanuel Dumas, Jeroen Raes, Jean-Michel Oppert, Ivica Letunic, Jens Nielsen, Peer Bork, S. Dusko Ehrlich, Michael Stumvoll, Oluf Pedersen, Judith Aron-Wisnewsky, Karine Clément, Fredrik Bäckhed

**Affiliations:** 1 Wallenberg Laboratory, Department of Molecular and Clinical Medicine and Sahlgrenska Center for Cardiovascular and Metabolic Research, University of Gothenburg, 413 45 Gothenburg, Sweden; 2 Department of Medicine, Sahlgrenska University Hospital, Gothenburg, Sweden; 3 INSERM, Nutrition and Obesities; Systemic Approaches (NutriOmics), Sorbonne Université, Paris, France; 4 Assistance Publique Hôpitaux de Paris, Pitie-Salpêtrière Hospital, Nutrition department, CRNH Ile de France, Paris, France; 5 Integromics Unit, Institute of Cardiometabolism and Nutrition, 75013 Paris, France; 6 Medical Department III - Endocrinology, Nephrology, Rheumatology, University of Leipzig Medical Center, Leipzig, Germany; 7 Novo Nordisk Foundation Center for Basic Metabolic Research, Faculty of Health and Medical Sciences, University of Copenhagen, Blegdamsvej 3B, 2200 Copenhagen, Denmark; 8 Assistance Publique Hôpitaux de Paris, Clinical Investigation Center Paris East, 75013 Paris, France; 9 Assistance Publique Hôpitaux de Paris, Biochemistry and Hormonology Department, Tenon Hospital, 75020 Paris, France; 10 Experimental and Clinical Research Center, A Cooperation of Charité-Universitätsmedizin and the Max-Delbrück Center, Berlin, Germany; 11 Micalis Institute, INRA, AgroParisTech, Université Paris-Saclay, Paris, France; 12 Laboratory of Molecular Bacteriology, Department of Microbiology and Immunology, Rega Institute, KU Leuven Leuven, Belgium; 13 Center for Microbiology, VIB Leuven, Belgium; 14 Unité de Modélisation Mathématique et Informatique des Systèmes Complexes, UMMISCO, 93143 Bondy, France; 15 Sorbonne Paris Cité Epidemiology and Statistics Research Centre (CRESS), U1153 Inserm, U1125, Inra, Cnam, University of Paris 13, Nutritional Epidemiology Research Team (EREN), 93017 Bobigny, France; 16 Computational and Systems Medicine, Department of Metabolism, Digestion and Reproduction, Faculty of Medicine, Imperial College London, London, SW7 2AZ UK; 17 Genomic and Environmental Medicine, National Heart & Lung Institute, Faculty of Medicine, Imperial College London, London, SW3 6KY UK; 18 Biobyte Solutions GmbH, Bothestr. 142, 69117 Heidelberg, Germany; 19 Department of Biology and Biological Engineering, Chalmers University of Technology, SE41128 Gothenburg, Sweden; 20 Structural and Computational Biology, European Molecular Biology Laboratory, Heidelberg, Germany; 21 Molecular Medicine Partnership Unit, University of Heidelberg and European Molecular Biology Laboratory, Heidelberg, Germany; 22 Department of Clinical Physiology, Region Västra Götaland, Sahlgrenska University Hospital, Gothenburg, Sweden; 23 Assistance Publique Hôpitaux de Paris, Diabetes Department, Pitie-Salpêtrière Hospital, Paris, France; 24 Assistance Publique Hôpitaux de Paris, Cardiology Department, Pitie-Salpêtrière Hospital, Paris, France; 25 INRAE, Metagenopolis, Université Paris-Saclay, Jouy en Josas, France; 26 Assistance Publique Hôpitaux de Paris, Pitie-Salpêtrière Hospital, Biochemistry Department of Metabolic Disorders, Paris, France; 27 Institute of Science and Technology for Brain-Inspired Intelligence, Fudan University, Shanghai, China; 28 Centre de Recherche Saint-Antoine, Sorbonne Université-INSERM UMR-S 938, IHU ICAN, Paris, France; 29 Assistance Publique Hôpitaux de Paris, Endocrinology Department, Pitie-Salpêtrière Hospital, Paris, France; 30 Department of Clinical Biochemistry, University of Copenhagen, Rigshospitalet, Copenhagen, Denmark; 31 Assistance Publique Hôpitaux de Paris, Department of Pharmacology, Pitie-Salpêtrière Hospital, NICO Cardio-oncology Program, CIC-1421, INSERM, Sorbonne Université, Paris, France; 32 PARCC, INSERM, Université de Paris, Paris, France; 33 Assistance Publique Hôpitaux de Paris, Hôpital Européen Georges-Pompidou, CIC1418 and DMU CARTE, Paris, France; 34 Integrative Phenomics, Paris, France

**Keywords:** Microbiology, Endocrinology

Correction to: *Nature Communications* 10.1038/s41467-020-19589-w, published online 18 November 2020.

The original version of this Article contained an error in the spelling of the author Judith Aron-Wisnewsky, which was incorrectly given as Judith Aron-Wisneswky. This has now been corrected in both the PDF and HTML versions of the Article.

