# Supplementary material for: Imidazole propionate is increased in diabetes and associated with dietary patterns and altered microbial ecology
Source: Nat Commun. 2020 Nov 18;11:5881. doi: 10.1038/s41467-020-19589-w (PMC7676231; doi:10.1038/s41467-020-19589-w)
Supplement: Supplementary file 1 — Supplementary Information [file 41467_2020_19589_MOESM1_ESM.pdf]

Supplementary information for

Imidazole propionate is increased in diabetes and associated with dietary patterns and altered microbial ecology

Molinaro A, Bel Lassen P, et al.

Supplementary Figure 1

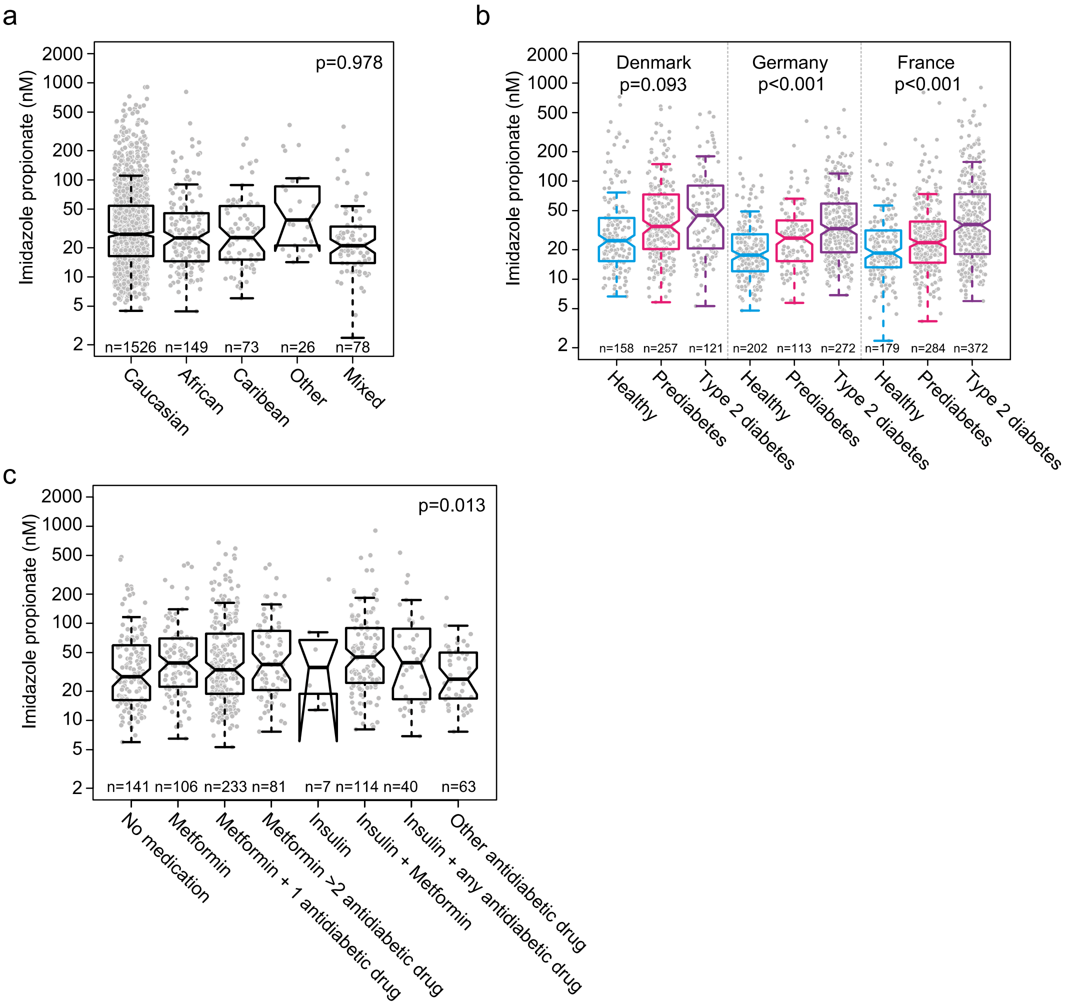

Supplementary Figure 1: Imidazole propionate levels according to ethnicity, enrolment center and antidiabetic treatment. Imidazole propionate according to (a) ethnicity (b) enrolment center and (c) type of antidiabetics drugs. p values were calculated with linear regression after adjustment for age, gender, BMI, ethnicity and creatinine clearance when applicable. Data are represented as boxplots: middle line is the median, the lower and upper hinges are the first and third quartiles, the upper whisker extends from the hinge to the largest value no further than 1.5 × the inter-quartile range (IQR) from the hinge and the lower whisker extends from the hinge to the smallest value at most 1.5 × IQR of the hinge. Grey dots are single data points. Source data are provided as a Source Data file.

Supplementary Figure 2

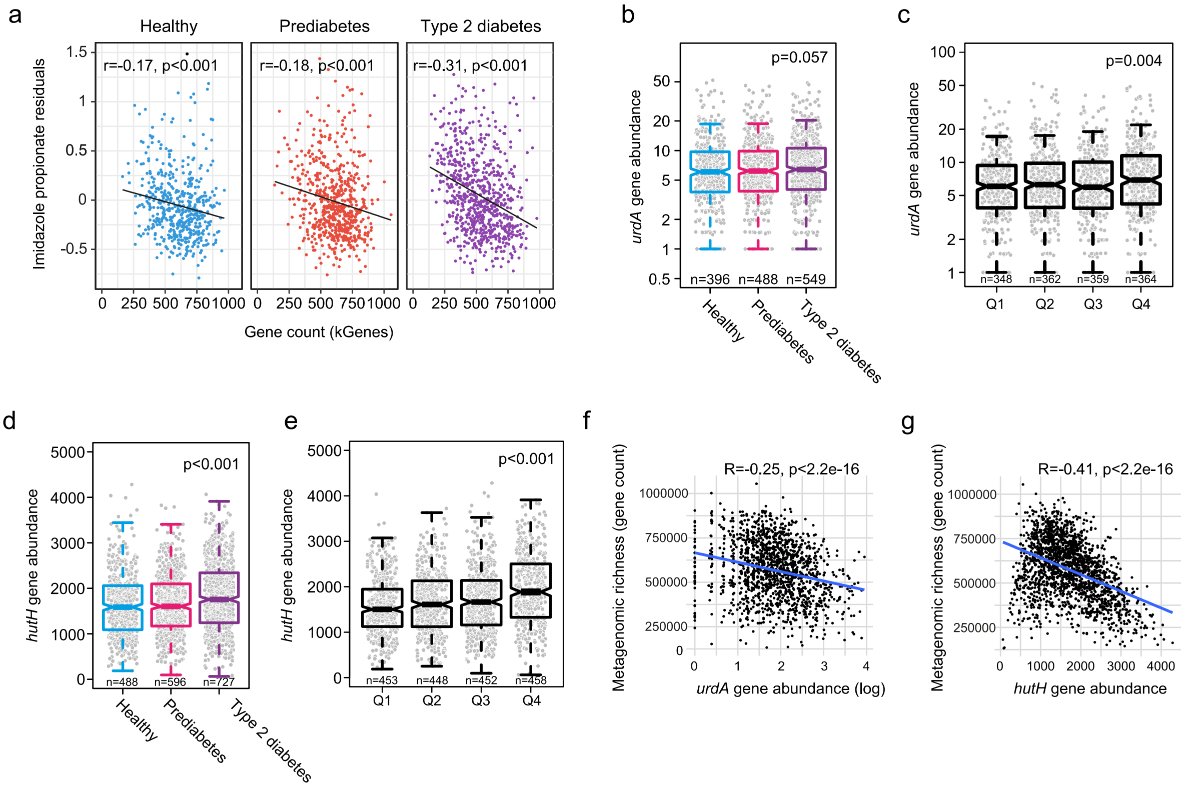

Supplementary Figure 2: Abundances of *hutH* and *urdA* are negatively correlated with metagenomic richness. a. Correlation analysis of gene count with imidazole propionate (ImP) residuals according to diabetes status. ImP residuals were calculated using a linear regression adjusted for age, gender, creatinine clearance, BMI and ethnicity. b-e. Relative abundance of *urdA* and *hutH* according to diabetes status and quartiles of serum ImP concentration. p values were calculated with linear regression after adjustment for age, gender, BMI, ethnicity, and creatinine clearance. Data are represented as boxplots: middle line is the median, the lower and upper hinges are the first and third quartiles, the upper whisker extends from the hinge to the largest value no further than 1.5 × the inter-quartile range (IQR) from the hinge and the lower whisker extends from the hinge to the smallest value at most 1.5 × IQR of the hinge. Grey dots are single data points. Source data are provided as a Source Data file. Log transformed relative abundance of (**f**) *urdA* and (**g**) *hutH* correlations with gene count. Correlations coefficients and p values were determined according to Spearman's method. Source data are provided as a Source Data file.

Supplementary Table 1

	Quartiles ImP	Prediabetes<br>Odds ratio (95% CI)	p value	Type 2 diabetes<br>Odds ratio (95% CI)	p value
Unadjusted	Q1 (<16.2)	reference	-	reference	-
	Q2 (13.3-27.2)	1.36 (1-1.84)	0.048	1.45 (1.07-1.97)	0.018
	Q3 (27.3-52.5)	2.40 (1.74-3.30)	<0.001	2.82 (2.05-3.89)	<0.001
	Q4 (52.7-901.2)	2.78 (1.96-3.93)	<0.001	5.08 (3.62-7.11)	<0.001
Model 1	Q1 (<16.2)	reference	-	reference	-
	Q2 (13.3-27.2)	1.04 (0.75-1.44)	0.832	1.03 (0.75-1.47)	0.873
	Q3 (27.3-52.5)	1.59 (1.12-2.25)	0.009	1.63 (1.13-2.37)	0.010
	Q4 (52.7-901.2)	1.70 (1.16-2.49)	0.006	2.50 (1.69-3.69)	<0.001
Model 2	Q1 (<16.2)	reference	-	reference	-
	Q2 (13.3-27.2)	1.05 (0.75-1.46)	0.986	1.07 (0.74-1.52)	0.697
	Q3 (27.3-52.5)	1.64 (1.15-2.32)	0.007	1.74 (1.19-2.53)	0.002
	Q4 (52.7-901.2)	1.75 (1.18-2.57)	0.006	2.76 (1.86-4.12)	<0.001

Supplementary Table 1: Multinominal logistic regression according to quartiles (Q) of imidazole propionate (ImP) for pre- and type 2 diabetes. p values were calculated using multinomial logistic regression model: unadjusted; Model 1 adjusted for age, gender, BMI, ethnicity; Model 2 adjusted for model 1 plus creatinine clearance.

Supplementary Table 2

Variables	Beta	p.value	q.value	Symbol	Partial correlation coefficient	Group
HbA1c	0.193264802	1.01E-17	6.08E-17	**	0.193264802	univariate
Glycemia	0.182722316	5.75E-16	1.72E-15	**	0.182722316	univariate
Insulinemia	0.150734631	3.21E-11	4.81E-11	**	0.150734631	univariate
HOMA B	-0.07630798	0.001710028	0.001710028	**	-0.07630798	univariate
HOMA IR	0.126928962	1.69E-07	2.03E-07	**	0.126928962	univariate
Tyg index	0.180890715	1.21E-15	2.41E-15	**	0.180890715	univariate
HbA1c	0.120266692	1.28E-07	7.69E-07	**	0.120266692	model1
Glycemia	0.074464563	0.001103456	0.001324147	**	0.074464563	model1
Insulinemia	0.10397807	5.49E-06	1.65E-05	**	0.10397807	model1
HOMA B	-<br>0.013925987	0.569215607	0.569215607	NA	-0.013925987	model1
HOMA IR	0.101783013	3.03E-05	6.07E-05	**	0.101783013	model1
Tyg index	0.082762463	0.000290413	0.00043562	**	0.082762463	model1
HbA1c	0.132472173	6.20E-09	3.72E-08	**	0.132472173	model2
Glycemia	0.088460231	0.000108352	0.000162528	**	0.088460231	model2
Insulinemia	0.108929562	2.02E-06	6.05E-06	**	0.108929562	model2
HOMA B	-<br>0.027212008	0.266966584	0.266966584	NA	-0.027212008	model2
HOMA IR	0.104699459	1.85E-05	3.70E-05	**	0.104699459	model2
Tyg index	0.086233494	0.000163049	0.000195658	**	0.086233494	model2
HbA1c	0.063645177	0.005405987	0.010811974	**	0.063645177	model3
Glycemia	0.017017798	0.457414966	0.457414966	NA	0.017017798	model3
Insulinemia	0.073162097	0.001445797	0.004337391	**	0.073162097	model3
HOMA B	0.019927996	0.416435872	0.457414966	NA	0.019927996	model3
HOMA IR	0.082409663	0.000763002	0.004337391	**	0.082409663	model3
Tyg index	0.034619375	0.130822661	0.196233991	NA	0.034619375	model3

Supplementary Table 2: Pearson partial correlation coefficients for imidazole propionate and markers of impaired glucose metabolism. *$p < 0.05$, ** False discovery rate (FDR) adjustment for $p < 0.05$

Supplementary Table 3

	Q1 (<16.2)	Q2 (13.3-27.2)	Q3 (27.3-52.5)	Q4 (52.7-901.2)	p value
n	135	145	149	157	
Glucose (nM)	6.3 (5.3-7.8)	6.2(5.3-7.7)	6.6(5.5-8.0)	6.6(5.5-8.5)	0.053
Insulin (mUI)	30.4 (21.4-55.4)	32 (18.5-62.10)	39.5 (22.4-64.9)	38.5 (23.8-62.8)	0.020
C-Peptide (ug/L)	7.1 (5.4-9.2)	7.3 (5.47-10.1)	8.1 (6.2-10.1)	8.3 (6.4-10.9)	0.00014
Stumvoll Sensitivity index	0.095 (0.076-0.104)	0.092 (0.072-0.107)	0.088 (0.058-0.103)	0.083 (0.064-0.102)	0.004

Supplementary Table 3: 2-hours serum glucose, insulin, C-peptide levels and Stumvoll Sensitivity Index after oral glucose tolerance according to quartiles levels of imidazole propionate according to imidazole propionate quartiles (Q). Data obtained from a subset of MetaCardis cohort (n=586). p values were calculated using linear regression model.

Supplementary Table 4

Variables	Increase in node purity	Correlation coefficient	p.value	q.value	Correlation with ImP
Coprococcus_comes	1.642573604	-0.111181168	1.82E-06	2.79E-06	negative
Dorea_formicigenerans	1.463595845	-0.086671259	0.000202257	0.00022473	negative
Dorea_longicatena	0.971225794	-0.048780758	0.036719761	0.03865238	negative
Eubacterium_eligens	1.037167562	-0.176461943	2.71E-14	7.74E-14	negative
Faecalibacterium_prausnitzii	1.483652523	-0.094512417	5.04E-05	5.93E-05	negative
Subdoligranulum_variabile	0.870215223	-0.047930151	0.040130833	0.040130833	negative
Bacteroides_dorei_vulgatus	0.9239209	0.144232609	5.47E-10	1.37E-09	positive
Bacteroides_ovatus	1.065662996	0.096007322	3.82E-05	5.09E-05	positive
Bacteroides_xylanisolvens	1.073375974	0.122428856	1.45E-07	2.41E-07	positive
Bifidobacterium_longum	1.198733	0.13406643	8.24E-09	1.65E-08	positive
Clostridium_bartlettii	1.032897892	0.095581541	4.13E-05	5.17E-05	positive
Clostridium_bolteae	2.755278513	0.200779801	<1E-16	<1E-16	positive
Clostridium_ramosum	1.319685722	0.209217719	<1E-16	<1E-16	positive
Clostridium_scindens	1.223055998	0.18799422	4.44E-16	2.22E-15	positive
Clostridium_symbiosum	1.513025804	0.179593848	9.33E-15	3.11E-14	positive
Eggerthella_lenta	0.908292124	0.181936489	4.00E-15	1.60E-14	positive
Pseudoflavonifractor_capillosus	1.127715688	0.13260629	1.20E-08	2.18E-08	positive
Ruminococcus_gnavus	2.150152475	0.229083504	<1E-16	<1E-16	positive
Veillonella_atypica	0.982732567	0.135342806	5.92E-09	1.32E-08	positive
Veillonella_parvula	1.071466862	0.109192132	2.77E-06	3.96E-06	positive

Supplementary Table 4: 20 most significant mOTUs correlated with imidazole propionate (ImP) residuals. p values were calculated using random forest models using ImP residuals adjusted for age, gender, BMI, diabetes status, ethnicity and creatinine clearance.

Supplementary Table 5

Variable	p.value	q.value	Symbol	Correlation coefficient	Model
Leucocytes	3.08E-06	3.08E-05	**	0.10555127	univariate
Neutrophiles (%)	0.00884815	0.01313202	**	0.05943668	univariate
Monocytes (%)	0.0210675	0.02633438	**	0.05238389	univariate
Lymphocytes (%)	6.44E-06	3.22E-05	**	-0.102249	univariate
CRP	0.00919241	0.01313202	**	0.05895879	univariate
IL6	0.00111824	0.00223648	**	0.07369097	univariate
IL7	0.33594449	0.37327166	NA	0.02201118	univariate
CXCL5	0.00100789	0.00223648	**	-0.0751252	univariate
CCL2	0.88309579	0.88309579	NA	0.00336417	univariate
IP10	0.00013878	0.00046259	**	0.08700918	univariate
Leucocytes	1.76E-05	0.00017647	**	0.09756958	model1
Neutrophiles (%)	0.41655824	0.41655824	NA	0.01853656	model1
Monocytes (%)	0.39312409	0.41655824	NA	-0.0194846	model1
Lymphocytes (%)	0.12307443	0.30768607	NA	-0.0351756	model1
CRP	0.01876555	0.06255185	*	0.0534228	model1
IL6	0.19412404	0.35365525	NA	0.02951581	model1
IL7	0.40102096	0.41655824	NA	0.01929247	model1
CXCL5	0.21219315	0.35365525	NA	-0.0286566	model1
CCL2	0.35167293	0.41655824	NA	0.02139516	model1
IP10	0.00937464	0.0468732	**	0.05963739	model1
Leucocytes	8.42E-06	8.42E-05	**	0.10140385	model2
Neutrophiles (%)	0.40779257	0.40779257	NA	0.01892187	model2
Monocytes (%)	0.40487431	0.40779257	NA	-0.0190399	model2
Lymphocytes (%)	0.1210296	0.30257399	NA	-0.0354333	model2
CRP	0.03197909	0.10659695	*	0.04887145	model2
IL6	0.40652149	0.40779257	NA	0.018914	model2
IL7	0.37371592	0.40779257	NA	0.02047806	model2
CXCL5	0.22959003	0.40779257	NA	-0.0276555	model2
CCL2	0.3250122	0.40779257	NA	0.02265707	model2
IP10	0.00835926	0.04179628	**	0.06066178	model2
Leucocytes	0.00018792	0.00187916	**	0.08511174	model3
Neutrophiles (%)	0.35921965	0.39894512	NA	0.0209629	model3
Monocytes (%)	0.39894512	0.39894512	NA	-0.0192862	model3
Lymphocytes (%)	0.07156601	0.14313202	NA	-0.0411849	model3
CRP	0.04675287	0.14313202	*	0.045329	model3
IL6	0.34379238	0.39894512	NA	0.02157755	model3
IL7	0.29801169	0.39894512	NA	0.02396364	model3
CXCL5	0.06806751	0.14313202	NA	-0.0420004	model3
CCL2	0.32574169	0.39894512	NA	0.02262894	model3
IP10	0.00584179	0.02920893	**	0.06341798	model3

Supplementary Table 5: Partial correlation matrix for imidazole propionate serum levels and serum inflammatory markers. *$p < 0.05$, ** $p < 0.01$. C-reactive protein (CRP), Interleukin 6 (IL6), Interleukin 7 (IL7), Interferon gamma-induced protein 10 (IP10), C-X-C motif chemokine 5 (CXCL5), chemokine (C-C motif) ligand 2 (CCL2)

Supplementary Table 6

Variable	p.value	q.value	Symbol	Correlation coefficient	Model
B Lymphocytes (%)	0.120215493	0.160287324	NA	-0.067514351	univariate
T Lymphocytes (%)	0.033761353	0.067522706	*	-0.092147541	univariate
TREG (%)	0.178289621	0.178289621	NA	0.068123836	univariate
MAIT (%)	0.001171391	0.004685563	**	-0.163354602	univariate
B Lymphocytes (%)	0.472175061	0.472175061	NA	-0.031565502	model1
T Lymphocytes (%)	0.157643112	0.236464668	NA	-0.06199588	model1
TREG (%)	0.121887816	0.236464668	NA	0.079281388	model1
MAIT (%)	0.00011929	0.00011929	**	-0.195595633	model1
B Lymphocytes (%)	0.929908301	0.929908301	NA	-0.003881631	model2
T Lymphocytes (%)	0.383894415	0.575841623	NA	-0.038411337	model2
TREG (%)	0.076412368	0.229237103	NA	0.091248324	model2
MAIT (%)	0.000276514	0.000276514	**	-0.186015512	model2
B Lymphocytes (%)	0.231749669	0.231749669	NA	-0.052787583	model3
T Lymphocytes (%)	0.167892937	0.231749669	NA	-0.060857804	model3
TREG (%)	0.050515821	0.151547462	NA	0.100795064	model3
MAIT (%)	0.002220664	0.002220664	**	-0.157089648	model3

Supplementary Table 6: Partial correlation matrix in a subgroup of patients (n=439) between serum imidazole propionate levels and white blood cells subtype. * $p<0.05$, ** $p<0.01$. Regulatory T cells (TREG, %) and mucosal associated invariant T cell (MAIT, %).

Supplementary Table 7

Co-abundance group	Species	Genus	Family	Order	Class	Phylum	Superkingdom
CAG00001	Unclassified	Unclassified	Unclassified	Unclassified	Unclassified	Unclassified	Unclassified
CAG00002	Unclassified	Unclassified	Unclassified	Unclassified	Unclassified	Unclassified	Unclassified
CAG00003	Unclassified	Unclassified	Unclassified	Unclassified	Unclassified	Unclassified	Unclassified
CAG00004	Blastocystis sp. ST4 (obsolete)	Blastocystis	unclassified Eukaryota	unclassified Eukaryota	unclassified Eukaryota	unclassified Eukaryota	Eukaryota
CAG00012	Klebsiella pneumoniae 1	Klebsiella	Enterobacteriaceae	Enterobacteriales	Gammaproteobacteria	Proteobacteria	Bacteria
CAG00024	Bacteroides fragilis 1	Bacteroides	Bacteroidaceae	Bacteroidales	Bacteroidia	Bacteroidetes	Bacteria
CAG00028	Klebsiella oxytoca	Klebsiella	Enterobacteriaceae	Enterobacteriales	Gammaproteobacteria	Proteobacteria	Bacteria
CAG01028	Ruminococcaceae bacterium LM158	unclassified Ruminococcaceae	Ruminococcaceae	Clostridiales	Clostridia	Firmicutes	Bacteria
CAG00030	Enterobacter aerogenes	Enterobacter	Enterobacteriaceae	Enterobacteriales	Gammaproteobacteria	Proteobacteria	Bacteria
CAG00034	Enterobacter cloacae 2	Enterobacter	Enterobacteriaceae	Enterobacteriales	Gammaproteobacteria	Proteobacteria	Bacteria
CAG00062	Bacteroides salyersiae	Bacteroides	Bacteroidaceae	Bacteroidales	Bacteroidia	Bacteroidetes	Bacteria
CAG00043	Enterobacter cloacae 3	Enterobacter	Enterobacteriaceae	Enterobacteriales	Gammaproteobacteria	Proteobacteria	Bacteria
CAG00044	Clostridium symbiosum	Lachnoclostridium	Lachnospiraceae	Clostridiales	Clostridia	Firmicutes	Bacteria
CAG00046	Unclassified	Bacteroides	Bacteroidaceae	Bacteroidales	Bacteroidia	Bacteroidetes	Bacteria
CAG00050	Bacteroides thetaiotaomicron	Bacteroides	Bacteroidaceae	Bacteroidales	Bacteroidia	Bacteroidetes	Bacteria
CAG00052	Parabacteroides goldsteinii	Parabacteroides	Porphyromonadaceae	Bacteroidales	Bacteroidia	Bacteroidetes	Bacteria
CAG00054	Unclassified	Unclassified	Unclassified	Unclassified	Unclassified	Firmicutes	Bacteria
CAG00058	Unclassified	Bacteroides	Bacteroidaceae	Bacteroidales	Bacteroidia	Bacteroidetes	Bacteria
CAG00059	Unclassified	Bacteroides	Bacteroidaceae	Bacteroidales	Bacteroidia	Bacteroidetes	Bacteria
CAG00061	Unclassified	Unclassified	Unclassified	Unclassified	Unclassified	Unclassified	Unclassified
CAG00066	Unclassified	Bacteroides	Bacteroidaceae	Bacteroidales	Bacteroidia	Bacteroidetes	Bacteria
CAG00146	Unclassified	Butyricimonas	Porphyromonadaceae	Bacteroidales	Bacteroidia	Bacteroidetes	Bacteria
CAG00071	Bacteroides cellulosilyticus	Bacteroides	Bacteroidaceae	Bacteroidales	Bacteroidia	Bacteroidetes	Bacteria
CAG00072	Odoribacter laneus	Odoribacter	Porphyromonadaceae	Bacteroidales	Bacteroidia	Bacteroidetes	Bacteria
CAG00073	Unclassified	Unclassified	Unclassified	Unclassified	Unclassified	Unclassified	Unclassified
CAG00074	Unclassified	Parabacteroides	Porphyromonadaceae	Bacteroidales	Bacteroidia	Bacteroidetes	Bacteria
CAG00075	Unclassified	Bacteroides	Bacteroidaceae	Bacteroidales	Bacteroidia	Bacteroidetes	Bacteria
CAG00076	Unclassified	Blautia	Lachnospiraceae	Clostridiales	Clostridia	Firmicutes	Bacteria
CAG00081	Bacteroides fragilis 2	Bacteroides	Bacteroidaceae	Bacteroidales	Bacteroidia	Bacteroidetes	Bacteria

CAG00083	Odoribacter sp. CAG:788	Odoribacter	Porphyromonadaceae	Bacteroidales	Bacteroidia	Bacteroidetes	Bacteria
CAG00085	Unclassified	Unclassified	Porphyromonadaceae	Bacteroidales	Bacteroidia	Bacteroidetes	Bacteria
CAG00086	Bacteroides massiliensis	Bacteroides	Bacteroidaceae	Bacteroidales	Bacteroidia	Bacteroidetes	Bacteria
CAG00087	Unclassified	Bacteroides	Bacteroidaceae	Bacteroidales	Bacteroidia	Bacteroidetes	Bacteria
CAG00089	Unclassified	Blautia	Lachnospiraceae	Clostridiales	Clostridia	Firmicutes	Bacteria
CAG00093	Klebsiella pneumoniae 2	Klebsiella	Enterobacteriaceae	Enterobacteriales	Gammaproteobacteria	Proteobacteria	Bacteria
CAG00098	Bacteroides fluxus	Bacteroides	Bacteroidaceae	Bacteroidales	Bacteroidia	Bacteroidetes	Bacteria
CAG00105	Unclassified	Bacteroides	Bacteroidaceae	Bacteroidales	Bacteroidia	Bacteroidetes	Bacteria
CAG00106	Unclassified	Prevotella	Prevotellaceae	Bacteroidales	Bacteroidia	Bacteroidetes	Bacteria
CAG00109	Unclassified	Fusobacterium	Fusobacteriaceae	Fusobacteriales	Fusobacteriia	Fusobacteria	Bacteria
CAG00111	Unclassified	Alistipes	Rikenellaceae	Bacteroidales	Bacteroidia	Bacteroidetes	Bacteria
CAG00116	Bacteroides nordii	Bacteroides	Bacteroidaceae	Bacteroidales	Bacteroidia	Bacteroidetes	Bacteria
CAG00117	Parabacteroides merdae	Parabacteroides	Porphyromonadaceae	Bacteroidales	Bacteroidia	Bacteroidetes	Bacteria
CAG00118	Bacteroides vulgatus	Bacteroides	Bacteroidaceae	Bacteroidales	Bacteroidia	Bacteroidetes	Bacteria
CAG00124	Klebsiella variicola	Klebsiella	Enterobacteriaceae	Enterobacteriales	Gammaproteobacteria	Proteobacteria	Bacteria
CAG00267	Clostridium sp. CAG:58	unclassified Clostridiales	unclassified Clostridiales	Clostridiales	Clostridia	Firmicutes	Bacteria
CAG00126	Parabacteroides gordonii	Parabacteroides	Porphyromonadaceae	Bacteroidales	Bacteroidia	Bacteroidetes	Bacteria
CAG00128	Unclassified	Blautia	Lachnospiraceae	Clostridiales	Clostridia	Firmicutes	Bacteria
CAG00129	Unclassified	Unclassified	Desulfovibrionaceae	Desulfovibrionales	Deltaproteobacteria	Proteobacteria	Bacteria
CAG00131	Bacteroides sp. CAG:754	Bacteroides	Bacteroidaceae	Bacteroidales	Bacteroidia	Bacteroidetes	Bacteria
CAG00133	Unclassified	Bacteroides	Bacteroidaceae	Bacteroidales	Bacteroidia	Bacteroidetes	Bacteria
CAG00134	Unclassified	Cloacibacillus	Synergistaceae	Synergistales	Synergistia	Synergistetes	Bacteria
CAG00135	Bacteroides eggerthii	Bacteroides	Bacteroidaceae	Bacteroidales	Bacteroidia	Bacteroidetes	Bacteria
CAG00384	Alistipes shahii	Alistipes	Rikenellaceae	Bacteroidales	Bacteroidia	Bacteroidetes	Bacteria
CAG00138	Unclassified	Parabacteroides	Porphyromonadaceae	Bacteroidales	Bacteroidia	Bacteroidetes	Bacteria
CAG00141	Parabacteroides distasonis	Parabacteroides	Porphyromonadaceae	Bacteroidales	Bacteroidia	Bacteroidetes	Bacteria
CAG00142	Bacteroides stercoris	Bacteroides	Bacteroidaceae	Bacteroidales	Bacteroidia	Bacteroidetes	Bacteria
CAG00143	Unclassified	unclassified Firmicutes	unclassified Firmicutes	unclassified Firmicutes	unclassified Firmicutes	Firmicutes	Bacteria
CAG00144	Fusobacterium ulcerans	Fusobacterium	Fusobacteriaceae	Fusobacteriales	Fusobacteriia	Fusobacteria	Bacteria
CAG00260	Acidaminococcus intestini	Acidaminococcus	Acidaminococcaceae	Selenomonadales	Negativicutes	Firmicutes	Bacteria
CAG00153	Hafnia paralvei	Hafnia	Enterobacteriaceae	Enterobacteriales	Gammaproteobacteria	Proteobacteria	Bacteria

CAG00155	Firmicutes bacterium CAG:424	unclassified Firmicutes	unclassified Firmicutes	unclassified Firmicutes	unclassified Firmicutes	Firmicutes	Bacteria
CAG00158	Unclassified	Bacteroides	Bacteroidaceae	Bacteroidales	Bacteroidia	Bacteroidetes	Bacteria
CAG00159	Bacteroides uniformis	Bacteroides	Bacteroidaceae	Bacteroidales	Bacteroidia	Bacteroidetes	Bacteria
CAG00163	Prevotella sp. CAG:255	Prevotella	Prevotellaceae	Bacteroidales	Bacteroidia	Bacteroidetes	Bacteria
CAG00172	Unclassified	Bacteroides	Bacteroidaceae	Bacteroidales	Bacteroidia	Bacteroidetes	Bacteria
CAG00175	Bacteroides clarus	Bacteroides	Bacteroidaceae	Bacteroidales	Bacteroidia	Bacteroidetes	Bacteria
CAG00005_1	Unclassified	Unclassified	Unclassified	Unclassified	Unclassified	Firmicutes	Bacteria
CAG00180	Unclassified	Bacteroides	Bacteroidaceae	Bacteroidales	Bacteroidia	Bacteroidetes	Bacteria
CAG00181	Unclassified	Unclassified	Unclassified	Unclassified	Unclassified	Unclassified	Unclassified
CAG00182	Unclassified	Bacteroides	Bacteroidaceae	Bacteroidales	Bacteroidia	Bacteroidetes	Bacteria
CAG00184	Bacteroides sp. CAG:462	Bacteroides	Bacteroidaceae	Bacteroidales	Bacteroidia	Bacteroidetes	Bacteria
CAG00185	Veillonella sp. CAG:933	Veillonella	Veillonellaceae	Selenomonadales	Negativicutes	Firmicutes	Bacteria
CAG00191	Fusobacterium varium	Fusobacterium	Fusobacteriaceae	Fusobacteriales	Fusobacteriia	Fusobacteria	Bacteria
CAG00192	Unclassified	Unclassified	Unclassified	Unclassified	Unclassified	Firmicutes	Bacteria
CAG00193	Unclassified	Unclassified	Unclassified	Unclassified	Unclassified	Firmicutes	Bacteria
CAG00199	uncultured Alistipes sp.	Alistipes	Rikenellaceae	Bacteroidales	Bacteroidia	Bacteroidetes	Bacteria
CAG00202	Parabacteroides johnsonii	Parabacteroides	Porphyromonadaceae	Bacteroidales	Bacteroidia	Bacteroidetes	Bacteria
CAG00206	Pyramidobacter piscolens	Pyramidobacter	Synergistaceae	Synergistales	Synergistia	Synergistetes	Bacteria
CAG00211	Firmicutes bacterium CAG:227	unclassified Firmicutes	unclassified Firmicutes	unclassified Firmicutes	unclassified Firmicutes	Firmicutes	Bacteria
CAG00213	Unclassified	Prevotella	Prevotellaceae	Bacteroidales	Bacteroidia	Bacteroidetes	Bacteria
CAG00214	Prevotella corporis	Prevotella	Prevotellaceae	Bacteroidales	Bacteroidia	Bacteroidetes	Bacteria
CAG00220	Unclassified	Bacteroides	Bacteroidaceae	Bacteroidales	Bacteroidia	Bacteroidetes	Bacteria
CAG00945	Bacteroides xylanisolvens	Bacteroides	Bacteroidaceae	Bacteroidales	Bacteroidia	Bacteroidetes	Bacteria
CAG00231	Odoribacter splanchnicus	Odoribacter	Porphyromonadaceae	Bacteroidales	Bacteroidia	Bacteroidetes	Bacteria
CAG00237	Unclassified	Unclassified	Unclassified	Unclassified	Unclassified	Unclassified	Unclassified
CAG00238	Clostridium saccharolyticum	Lachnoclostridium	Lachnospiraceae	Clostridiales	Clostridia	Firmicutes	Bacteria
CAG00252	Unclassified	Sutterella	Sutterellaceae	Burkholderiales	Betaproteobacteria	Proteobacteria	Bacteria
CAG00263	Klebsiella oxytoca	Klebsiella	Enterobacteriaceae	Enterobacteriales	Gammaproteobacteria	Proteobacteria	Bacteria
CAG00280	Prevotella sp. CAG:485	Prevotella	Prevotellaceae	Bacteroidales	Bacteroidia	Bacteroidetes	Bacteria
CAG00287	Prevotella sp. CAG:1124	Prevotella	Prevotellaceae	Bacteroidales	Bacteroidia	Bacteroidetes	Bacteria

CAG00292	Unclassified	Unclassified	Unclassified	Unclassified	Unclassified	Unclassified	Unclassified
CAG00293	Firmicutes bacterium CAG:145	unclassified Firmicutes	unclassified Firmicutes	unclassified Firmicutes	unclassified Firmicutes	Firmicutes	Bacteria
CAG00298	Bacteroides intestinalis 1	Bacteroides	Bacteroidaceae	Bacteroidales	Bacteroidia	Bacteroidetes	Bacteria
CAG00299	Unclassified	Unclassified	Unclassified	Unclassified	Unclassified	Proteobacteria	Bacteria
CAG00309	Alistipes onderdonkii	Alistipes	Rikenellaceae	Bacteroidales	Bacteroidia	Bacteroidetes	Bacteria
CAG00310	Sutterella parvirubra	Sutterella	Sutterellaceae	Burkholderiales	Betaproteobacteria	Proteobacteria	Bacteria
CAG00312	Unclassified	Unclassified	Unclassified	Unclassified	Unclassified	Bacteroidetes	Bacteria
CAG00314	Unclassified	Alistipes	Rikenellaceae	Bacteroidales	Bacteroidia	Bacteroidetes	Bacteria
CAG00315	Citrobacter freundii	Citrobacter	Enterobacteriaceae	Enterobacteriales	Gammaproteobacteria	Proteobacteria	Bacteria
CAG00319	Unclassified	Unclassified	Unclassified	Unclassified	Unclassified	Unclassified	Unclassified
CAG00321	Clostridium sp. CAG:138	unclassified Clostridiales	unclassified Clostridiales	Clostridiales	Clostridia	Firmicutes	Bacteria
CAG00325	Unclassified	Parasutterella	Sutterellaceae	Burkholderiales	Betaproteobacteria	Proteobacteria	Bacteria
CAG00332	Unclassified	Parabacteroides	Porphyromonadaceae	Bacteroidales	Bacteroidia	Bacteroidetes	Bacteria
CAG00334	Prevotella sp. CAG:1092	Prevotella	Prevotellaceae	Bacteroidales	Bacteroidia	Bacteroidetes	Bacteria
CAG00019_1	Intestinibacter bartlettii	Intestinibacter	Peptostreptococcaceae	Clostridiales	Clostridia	Firmicutes	Bacteria
CAG00340	Unclassified	unclassified Clostridiales	unclassified Clostridiales	Clostridiales	Clostridia	Firmicutes	Bacteria
CAG00344	Unclassified	Unclassified	Unclassified	Bacteroidales	Bacteroidia	Bacteroidetes	Bacteria
CAG00352	Unclassified	Acidaminococcus	Acidaminococcaceae	Selenomonadales	Negativicutes	Firmicutes	Bacteria
CAG00355	Bacteroides sp. CAG:661	Bacteroides	Bacteroidaceae	Bacteroidales	Bacteroidia	Bacteroidetes	Bacteria
CAG00357	Bacteroides ovatus	Bacteroides	Bacteroidaceae	Bacteroidales	Bacteroidia	Bacteroidetes	Bacteria
CAG00360	Alistipes finegoldii	Alistipes	Rikenellaceae	Bacteroidales	Bacteroidia	Bacteroidetes	Bacteria
CAG00361	Unclassified	Acidaminococcus	Acidaminococcaceae	Selenomonadales	Negativicutes	Firmicutes	Bacteria
CAG00364	Blautia hansenii	Blautia	Lachnospiraceae	Clostridiales	Clostridia	Firmicutes	Bacteria
CAG00367	Firmicutes bacterium CAG:170	unclassified Firmicutes	unclassified Firmicutes	unclassified Firmicutes	unclassified Firmicutes	Firmicutes	Bacteria
CAG00368	Unclassified	Alistipes	Rikenellaceae	Bacteroidales	Bacteroidia	Bacteroidetes	Bacteria
CAG00370	Unclassified	Unclassified	Unclassified	Bacteroidales	Bacteroidia	Bacteroidetes	Bacteria
CAG00371	Bacteroides dorei	Bacteroides	Bacteroidaceae	Bacteroidales	Bacteroidia	Bacteroidetes	Bacteria
CAG00373	Unclassified	Unclassified	Unclassified	Clostridiales	Clostridia	Firmicutes	Bacteria
CAG00375	Unclassified	Prevotella	Prevotellaceae	Bacteroidales	Bacteroidia	Bacteroidetes	Bacteria
CAG00381	Clostridium sp. CAG:242	unclassified Clostridiales	unclassified Clostridiales	Clostridiales	Clostridia	Firmicutes	Bacteria
CAG00401	Prevotella sp. CAG:592	Prevotella	Prevotellaceae	Bacteroidales	Bacteroidia	Bacteroidetes	Bacteria

CAG00405	Unclassified	unclassified Firmicutes	unclassified Firmicutes	unclassified Firmicutes	unclassified Firmicutes	Firmicutes	Bacteria
CAG00412	Unclassified	Prevotella	Prevotellaceae	Bacteroidales	Bacteroidia	Bacteroidetes	Bacteria
CAG00416	Lelliottia amnigena	Lelliottia	Enterobacteriaceae	Enterobacteriales	Gammaproteobacteria	Proteobacteria	Bacteria
CAG00421	Unclassified	Alistipes	Rikenellaceae	Bacteroidales	Bacteroidia	Bacteroidetes	Bacteria
CAG00424	Firmicutes bacterium CAG:137	unclassified Firmicutes	unclassified Firmicutes	unclassified Firmicutes	unclassified Firmicutes	Firmicutes	Bacteria
CAG00436	Clostridium sp. CAG:299	unclassified Clostridiales	unclassified Clostridiales	Clostridiales	Clostridia	Firmicutes	Bacteria
CAG00016_2	Unclassified	Prevotella	Prevotellaceae	Bacteroidales	Bacteroidia	Bacteroidetes	Bacteria
CAG00458	Fusobacterium nucleatum	Fusobacterium	Fusobacteriaceae	Fusobacteriales	Fusobacteriia	Fusobacteria	Bacteria
CAG00467	Prevotella sp. CAG:873	Prevotella	Prevotellaceae	Bacteroidales	Bacteroidia	Bacteroidetes	Bacteria
CAG00479	Unclassified	Alistipes	Rikenellaceae	Bacteroidales	Bacteroidia	Bacteroidetes	Bacteria
CAG00489	Unclassified	Unclassified	Unclassified	Unclassified	Unclassified	Unclassified	Unclassified
CAG00497	Unclassified	unclassified Firmicutes	unclassified Firmicutes	unclassified Firmicutes	unclassified Firmicutes	Firmicutes	Bacteria
CAG00499	Prevotella sp. CAG:891	Prevotella	Prevotellaceae	Bacteroidales	Bacteroidia	Bacteroidetes	Bacteria
CAG00505	Unclassified	Unclassified	Unclassified	Unclassified	Unclassified	Firmicutes	Bacteria
CAG00510	Unclassified	Alistipes	Rikenellaceae	Bacteroidales	Bacteroidia	Bacteroidetes	Bacteria
CAG00521	Prevotella sp. CAG:1058	Prevotella	Prevotellaceae	Bacteroidales	Bacteroidia	Bacteroidetes	Bacteria
CAG00529	Prevotella sp. CAG:1185	Prevotella	Prevotellaceae	Bacteroidales	Bacteroidia	Bacteroidetes	Bacteria
CAG00533	Unclassified	Unclassified	Unclassified	Unclassified	Unclassified	Firmicutes	Bacteria
CAG00549	Bifidobacterium longum	Bifidobacterium	Bifidobacteriaceae	Bifidobacteriales	Actinobacteria	Actinobacteria	Bacteria
CAG00578	Alistipes putredinis	Alistipes	Rikenellaceae	Bacteroidales	Bacteroidia	Bacteroidetes	Bacteria
CAG00586	Unclassified	Blautia	Lachnospiraceae	Clostridiales	Clostridia	Firmicutes	Bacteria
CAG00596	Unclassified	unclassified Clostridiales	unclassified Clostridiales	Clostridiales	Clostridia	Firmicutes	Bacteria
CAG00600	Prevotella sp. CAG:1031	Prevotella	Prevotellaceae	Bacteroidales	Bacteroidia	Bacteroidetes	Bacteria
CAG00608	Unclassified	Alistipes	Rikenellaceae	Bacteroidales	Bacteroidia	Bacteroidetes	Bacteria
CAG00609	Bacteroides sp. CAG:770	Bacteroides	Bacteroidaceae	Bacteroidales	Bacteroidia	Bacteroidetes	Bacteria
CAG00629	Firmicutes bacterium CAG:124	unclassified Firmicutes	unclassified Firmicutes	unclassified Firmicutes	unclassified Firmicutes	Firmicutes	Bacteria
CAG00634	Unclassified	Prevotella	Prevotellaceae	Bacteroidales	Bacteroidia	Bacteroidetes	Bacteria
CAG00642	Unclassified	Prevotella	Prevotellaceae	Bacteroidales	Bacteroidia	Bacteroidetes	Bacteria
CAG00647	Unclassified	Sutterella	Sutterellaceae	Burkholderiales	Betaproteobacteria	Proteobacteria	Bacteria
CAG00655	Unclassified	Alistipes	Rikenellaceae	Bacteroidales	Bacteroidia	Bacteroidetes	Bacteria
CAG00660	Alistipes timonensis	Alistipes	Rikenellaceae	Bacteroidales	Bacteroidia	Bacteroidetes	Bacteria

CAG00664	Alistipes sp. CAG:268	Alistipes	Rikenellaceae	Bacteroidales	Bacteroidia	Bacteroidetes	Bacteria
CAG00672	Clostridium sp. CAG:169	unclassified Clostridiales	unclassified Clostridiales	Clostridiales	Clostridia	Firmicutes	Bacteria
CAG00694	Unclassified	Unclassified	Unclassified	Bacteroidales	Bacteroidia	Bacteroidetes	Bacteria
CAG00699	Veillonella ratti	Veillonella	Veillonellaceae	Selenomonadales	Negativicutes	Firmicutes	Bacteria
CAG00701	Unclassified	Bacteroides	Bacteroidaceae	Bacteroidales	Bacteroidia	Bacteroidetes	Bacteria
CAG00705	Prevotella sp. CAG:520	Prevotella	Prevotellaceae	Bacteroidales	Bacteroidia	Bacteroidetes	Bacteria
CAG00712	Unclassified	Unclassified	Unclassified	Unclassified	Unclassified	Proteobacteria	Bacteria
CAG00731	Parasutterella excrementihominis 2	Parasutterella	Sutterellaceae	Burkholderiales	Betaproteobacteria	Proteobacteria	Bacteria
CAG00734	Bacteroides sp. CAG:545	Bacteroides	Bacteroidaceae	Bacteroidales	Bacteroidia	Bacteroidetes	Bacteria
CAG00737	Unclassified	Alistipes	Rikenellaceae	Bacteroidales	Bacteroidia	Bacteroidetes	Bacteria
CAG00739	Unclassified	Unclassified	Unclassified	Unclassified	Unclassified	Unclassified	Unclassified
CAG00751	Firmicutes bacterium CAG:238	unclassified Firmicutes	unclassified Firmicutes	unclassified Firmicutes	unclassified Firmicutes	Firmicutes	Bacteria
CAG00961	Unclassified	Ruminococcaceae bacterium LM158	unclassified Ruminococcaceae	Ruminococcaceae	Clostridiales	Clostridia	Firmicutes
CAG00757	Bacteroides sp. CAG:709	Bacteroides	Bacteroidaceae	Bacteroidales	Bacteroidia	Bacteroidetes	Bacteria
CAG00798	Unclassified	Unclassified	Unclassified	Unclassified	Unclassified	Unclassified	Unclassified
CAG00802	Unclassified	Cloacibacillus	Synergistaceae	Synergistales	Synergistia	Synergistetes	Bacteria
CAG00810	Unclassified	Unclassified	Unclassified	Bacteroidales	Bacteroidia	Bacteroidetes	Bacteria
CAG00819	Acidaminococcus sp. CAG:542	Acidaminococcus	Acidaminococcaceae	Selenomonadales	Negativicutes	Firmicutes	Bacteria
CAG00836	Fusobacterium gonidiaformans	Fusobacterium	Fusobacteriaceae	Fusobacteriales	Fusobacteriia	Fusobacteria	Bacteria
CAG00849	Unclassified	Unclassified	Unclassified	Clostridiales	Clostridia	Firmicutes	Bacteria
CAG00853	Unclassified	Unclassified	Unclassified	Bacteroidales	Bacteroidia	Bacteroidetes	Bacteria
CAG00858	Streptococcus thermophilus	Streptococcus	Streptococcaceae	Lactobacillales	Bacilli	Firmicutes	Bacteria
CAG00860	Unclassified	Unclassified	Unclassified	Unclassified	Unclassified	Unclassified	Unclassified
CAG00862	Unclassified	Unclassified	Unclassified	Unclassified	Unclassified	Unclassified	Unclassified
CAG00873	Unclassified	Butyricimonas	Porphyromonadaceae	Bacteroidales	Bacteroidia	Bacteroidetes	Bacteria
CAG00878	Citrobacter koseri	Citrobacter	Enterobacteriaceae	Enterobacteriales	Gammaproteobacteria	Proteobacteria	Bacteria
CAG00883	Escherichia coli	Escherichia	Enterobacteriaceae	Enterobacteriales	Gammaproteobacteria	Proteobacteria	Bacteria
CAG00887	Alistipes sp. CAG:435	Alistipes	Rikenellaceae	Bacteroidales	Bacteroidia	Bacteroidetes	Bacteria
CAG00899	Unclassified	Alistipes	Rikenellaceae	Bacteroidales	Bacteroidia	Bacteroidetes	Bacteria
CAG00908	Unclassified	Intestinibacter	Peptostreptococcaceae	Clostridiales	Clostridia	Firmicutes	Bacteria

CAG00909	Parasutterella excrementihominis 1	Parasutterella	Sutterellaceae	Burkholderiales	Betaproteobacteria	Proteobacteria	Bacteria
CAG00942	Prevotella disiens	Prevotella	Prevotellaceae	Bacteroidales	Bacteroidia	Bacteroidetes	Bacteria
CAG00977	Unclassified	Unclassified	Unclassified	Unclassified	Unclassified	Unclassified	Unclassified
CAG01026	Peptoniphilus harei	Peptoniphilus	Peptoniphilaceae	Clostridiales	Clostridia	Firmicutes	Bacteria
CAG01069	Unclassified	Unclassified	Unclassified	Unclassified	Unclassified	Unclassified	Unclassified
CAG01127	Unclassified	Prevotella	Prevotellaceae	Bacteroidales	Bacteroidia	Bacteroidetes	Bacteria
CAG01130	Prevotella bivia	Prevotella	Prevotellaceae	Bacteroidales	Bacteroidia	Bacteroidetes	Bacteria
CAG01145	Unclassified	Bacteroides	Bacteroidaceae	Bacteroidales	Bacteroidia	Bacteroidetes	Bacteria
CAG01165	common Bacteroides xylanisolvens & ovatus	Bacteroides	Bacteroidaceae	Bacteroidales	Bacteroidia	Bacteroidetes	Bacteria
CAG01190	Butyricimonas virosa	Butyricimonas	Porphyromonadaceae	Bacteroidales	Bacteroidia	Bacteroidetes	Bacteria
CAG01195	Unclassified	Ruminococcaceae bacterium LM158	unclassified Ruminococcaceae	Ruminococcaceae	Clostridiales	Clostridia	Firmicutes
CAG00006_3	Unclassified	Oscillibacter	Oscillospiraceae	Clostridiales	Clostridia	Firmicutes	Bacteria
CAG01273	Enterobacter hormaechei	Enterobacter	Enterobacteriaceae	Enterobacteriales	Gammaproteobacteria	Proteobacteria	Bacteria
CAG01278	Unclassified	unclassified Firmicutes	unclassified Firmicutes	unclassified Firmicutes	unclassified Firmicutes	Firmicutes	Bacteria
CAG01289	Unclassified	Alistipes	Rikenellaceae	Bacteroidales	Bacteroidia	Bacteroidetes	Bacteria
CAG01339	Peptoniphilus lacrimalis	Peptoniphilus	Peptoniphilaceae	Clostridiales	Clostridia	Firmicutes	Bacteria
CAG01342	Unclassified	Ruminococcaceae bacterium LM158	unclassified Ruminococcaceae	Ruminococcaceae	Clostridiales	Clostridia	Firmicutes
CAG01368	Unclassified	Unclassified	Unclassified	Bacteroidales	Bacteroidia	Bacteroidetes	Bacteria
CAG01383	Unclassified	Prevotella	Prevotellaceae	Bacteroidales	Bacteroidia	Bacteroidetes	Bacteria
CAG01394	Unclassified	Blautia	Lachnospiraceae	Clostridiales	Clostridia	Firmicutes	Bacteria

Supplementry Table 7: List of metagenomic species with *hutH* gene

Supplementary Table 8

Variable	Beta	p.value	q.value	Symbol	Partial correlation coefficient
Red meat	-0.048231823	0.054272277	0.090453795	NA	-0.048231823
Poultry	-0.053262171	0.033531458	0.083828644	*	-0.053262171
Fish	-0.030800834	0.219198624	0.27399828	NA	-0.030800834
Milk	0.045786356	0.067704697	0.101557046	NA	0.045786356
Cheese	0.063674572	0.011022329	0.055111643	*	0.063674572
Yogurt	0.012433233	0.619986229	0.66427096	NA	0.012433233
Eggs	0.015007503	0.549473646	0.634008053	NA	0.015007503
Starch	-0.001099461	0.965025799	0.965025799	NA	-0.001099461
Vegetables	-0.055208206	0.027563531	0.082690593	*	-0.055208206
Fruits	-0.05057615	0.043557643	0.089518713	*	-0.05057615
Nuts	-0.090414244	0.000302418	0.004536277	**	-0.090414244
Energy intake	-0.020608008	0.410954473	0.513693091	NA	-0.020608008
Carbohydrate	0.031437497	0.209816158	0.349693597	NA	0.031437497
Sugar	0.005947827	0.812495558	0.812495558	NA	0.005947827
Fibre	-0.054125575	0.030759543	0.117435205	*	-0.054125575
Fat	-0.008905001	0.722478691	0.802754101	NA	-0.008905001
Unsaturated fat	-0.063547293	0.011183934	0.111839337	*	-0.063547293
Saturated fat	0.052762661	0.035230561	0.117435205	*	0.052762661
Protein	-0.043095393	0.085525298	0.213813244	NA	-0.043095393
Animal protein	-0.024239688	0.333620594	0.476600849	NA	-0.024239688
Vegetal protein	-0.033820829	0.177273707	0.349693597	NA	-0.033820829
aHEI score	-0.079267542	0.001544139	0.011581041	**	-0.079267542
DASH score	-0.04442065	0.076325173	0.104079782	NA	-0.04442065
DDS score	-0.049608171	0.047743314	0.089518713	*	-0.049608171
Med Diet Score	-0.060186549	0.016284221	0.06106583	*	-0.060186549

Supplementary Table 8: Pearson partial correlation coefficients for imidazole propionate and dietary nutrients and scores. p values were calculated using partial correlations adjusted for: age, gender, body mass index, ethnicity, diabetes status, creatinine clearance, daily energy intake (kcal/day) and enrolment center. * $p < 0.05$, ** False discovery rate (FDR) adjusted $p < 0.05$.
